# Impact of Excipients on Stability of Polymer Microparticles for Autoimmune Therapy

**DOI:** 10.3389/fbioe.2020.609577

**Published:** 2021-02-11

**Authors:** Emily A. Gosselin, Maeesha Noshin, Sheneil K. Black, Christopher M. Jewell

**Affiliations:** ^1^Fischell Department of Bioengineering, University of Maryland, College Park, College Park, MD, United States; ^2^Robert E Fischell Institute of Biomedical Devices, University of Maryland, College Park, College Park, MD, United States; ^3^United States Department of Veterans Affairs, Baltimore, MD, United States; ^4^Department of Microbiology and Immunology, University of Maryland Medical School, Baltimore, MD, United States; ^5^Marlene and Stewart Greenebaum Cancer Center, Baltimore, MD, United States

**Keywords:** stability, autoimmunity, immunotherapy, excipient, lyophilization, multiple sclerosis, formulation, nanotechnology

## Abstract

Therapies for autoimmune diseases such as multiple sclerosis and diabetes are not curative and cause significant challenges for patients. These include frequent, continued treatments required throughout the lifetime of the patient, as well as increased vulnerability to infection due to the non-specific action of therapies. Biomaterials have enabled progress in antigen-specific immunotherapies as carriers and delivery vehicles for immunomodulatory cargo. However, most of this work is in the preclinical stage, where small dosing requirements allow for on-demand preparation of immunotherapies. For clinical translation of these potential immunotherapies, manufacturing, preservation, storage, and stability are critical parameters that require greater attention. Here, we tested the stabilizing effects of excipients on the lyophilization of polymeric microparticles (MPs) designed for autoimmune therapy; these MPs are loaded with peptide self-antigen and a small molecule immunomodulator. We synthesized and lyophilized particles with three clinically relevant excipients: mannitol, trehalose, and sucrose. The biophysical properties of the formulations were assessed as a function of excipient formulation and stage of addition, then formulations were evaluated in primary immune cell culture. From a manufacturing perspective, excipients improved caking of lyophilized product, enabled more complete resuspension, increased product recovery, and led to smaller changes in MP size and size distribution over time. Cocultures of antigen-presenting cells and self-reactive T cells revealed that MPs lyophilized with excipients maintained tolerance-inducing function, even after significant storage times without refrigeration. These data demonstrate that excipients can be selected to drive favorable manufacturing properties without impacting the immunologic properties of the tolerogenic MPs.

## Introduction

Polymeric particles and scaffolds have been widely researched as carriers for biologics and small molecules to improve delivery of cargo to cell and tissue targets (Nair and Laurencin, 2007; Ulery et al., 2011; Danhier et al., 2012; Fenton et al., 2018). One important emerging area applies these carriers to immunotherapies by delivering peptides recognized by the immune system (antigens) and immune cues to modulate immune response (Andorko et al., 2015; Northrup et al., 2016; Tostanoski et al., 2016b; Bookstaver et al., 2018; Ben-Akiva et al., 2019). This development is motivated by the unique benefits of polymeric carriers and other designed materials in the context of immunotherapy, including co-delivery of immune signals, controlled release, prolonged cargo exposure, cargo protection, and preferential delivery to target immune cells (Tostanoski et al., 2016b; Bookstaver et al., 2018). While incorporating biomaterial carriers into these exciting new therapies adds potential, these components also add complexity for manufacturing and regulatory characterization. These are important aspects to address at an early stage to support more efficient translation of new immunotherapies.

We and others have recently explored the control that polymer carriers provide to correct the excessive inflammation and immune defects that occur in autoimmune disease such as multiple sclerosis (MS; Tostanoski et al., 2016b; Pearson et al., 2017; Gosselin et al., 2018; Dellacherie et al., 2019). MS, for example, occurs when the body’s immune system incorrectly attacks myelin, the matrix that surrounds neurons in the central nervous system (CNS). This attack is driven by myelin-specific immune cells, resulting in inflammation, neurodegeneration, and demyelination in the CNS (Bitsch et al., 2000; Sospedra and Martin, 2005; Comabella and Khoury, 2012). MS is treated with disease-modifying therapies that have improved patient quality of life but that are non-curative and non-specific. Even the newest monoclonal antibodies do not differentiate between normal cells and dysfunctional myelin-reactive immune cells or lymphocytes; this inhibitory function can leave patients susceptible to infection (Cross and Naismith, 2014). Therefore, an important goal for next-generation therapies is the induction of antigen-specific tolerance—correcting defects that occur in autoimmune disease without impacting normal immune responses. For example, using a simple, easily scalable degradable polymer [poly(lactide-co-glycolide), PLGA], we developed microparticle (MP) depots that promote regulatory immune function and reverse disease in preclinical models of MS. These depots recondition the local environment of lymph nodes—tissues that coordinate immune function—by co-delivering myelin self-peptide (MOG) and an immunomodulatory cue, rapamycin (Rapa). This shifts the response to myelin away from inflammation and toward tolerance in a selective fashion (Tostanoski et al., 2016a).

While these and other preclinical studies often afford the ability to make and begin testing immunotherapies on demand, translating candidate therapies require additional considerations to chemistry and manufacturing controls (CMCs), stability, and characterization after storage. These are all crucial aspects for regulatory approval but receive little attention in the drug delivery field relative to the number of preclinical therapeutic studies. Even well-established materials such as PLGA have ongoing issues for clinical translation in therapeutic contexts. While PLGA degrades by hydrolysis and is commonly used clinically, such as in degradable sutures, the degradation products of PLGA (e.g., lactic acid, glycolic acid) can be metabolized by cells (Danhier et al., 2012; Mohammadi-Samani and Taghipour, 2015). Further, any aqueous storage of particles requires consideration of unwanted cargo release, particle aggregation and fusion, and maintenance of cargo integrity during storage (Fonte et al., 2016b). Thus, the stability, transport, and storage of immunotherapeutic particles formed from degradable biopolymers are an important focus area.

As the pharmaceutical industry has long shown, freeze-drying (lyophilization)—a controlled dehydration process—is a common route to support particle storage that stabilizes particles and reduces degradation and aggregation. Lyophilization dehydrates particle suspensions via three steps: (1) freezing, (2) water removal by sublimation, and (3) desorption of unfrozen water by vacuum (Fonte et al., 2016b). However, polymer particles are complex, and their structure is susceptible to damage from the stresses of freeze-drying (Holzer et al., 2009; Fonte et al., 2016b), as well as the risks to the sensitive cargos that incorporated in these cargos as part of many immunotherapies. To limit this stress, cryoprotectants and lyoprotectants, including sugars such as mannitol, sucrose, and trehalose, can be added to alter the glass transition temperature of polymers and allow for a higher freezing rate (Holzer et al., 2009; Niu and Panyam, 2017; Shaikh et al., 2017; Chishti et al., 2019). These changes to the lyophilization process hinder ice crystal formation and limit mechanical stresses on particles, which results in better resuspension of the particles (Lee et al., 2009; Fonte et al., 2014, 2015, 2016a,b). The resulting glassy matrix also immobilizes MPs to protect from aggregation and degradation (Fonte et al.,2016a,b). Further, in the context of immunotherapies aimed at autoimmunity, these issues are crucial since the immune system amplifies responses; this means the homogeneity and stability of therapies that rely on particle delivery have an equally important role in determining efficacy and safety.

Although PLGA particles have been extensively characterized, there are knowledge gaps to address for immunotherapies to ensure that CMC considerations (e.g., lyophilization, long-term storage) do not impact integrity and cargo immune function during translation. To study the role of sugars as cryoprotectants in the freeze-drying of PLGA MP depots co-loaded with the candidate autoimmune therapeutic components mentioned above, we prepared samples of MPs loaded with MOG and Rapa, along with cryoprotectants. These cryoprotectants included mannitol, sucrose, or trehalose that were either encapsulated (Enc) in the MP depots during particle synthesis, or incorporated externally by resuspending in an aqueous solution with the cryoprotectant. The therapeutic MPs were then lyophilized and characterized to assess the long-term particle stability and cargo functionality following lyophilization and storage at room temperature. In this study, we show that low concentrations of Enc and externally incorporated (Ext) excipients enhance the stability of MPs after 5 months in storage at room temperature. MPs loaded with low concentrations of excipients were reconstituted more effectively than MPs without excipients and showed a smaller increase in size after storage than MPs with no excipients. In addition, MPs containing excipients maintained cargo biofunctionality: all of these MPs containing Rapa constrained immune cell activation and proliferation of myelin-specific T cells.

## Materials and Methods

### Materials

50:50 poly(lactide-co-glycolide) (inherent viscosity range: 0.55–0.75 dL/g) was purchased from Durect Corporation (Birmingham, AL, United States). High molecular weight poly(vinyl alcohol) (PVA) was purchased from Alfa Aesar (Tewksbury, MA, United States). Dichloromethane, mannitol, sucrose, and trehalose were purchased from Sigma Aldrich (St. Louis, MO, United States). Dimethyl sulfoxide was purchased from Thermo Fisher Scientific (Waltham, MA, United States). Myelin oligodendrocyte glycoprotein peptide (MOG_35__–__55_, MEVGWYRSPFSRVVHLYRNGK) was synthesized by Genscript (Piscataway, NJ, United States) with ≥98% purity, and Rapa was obtained from LC Laboratories (Woburn, MA, United States).

### Preparation of PLGA Microparticles

#### Synthesis of MPs

Microparticles were synthesized by double emulsion and solvent evaporation as previously described (Tostanoski et al., 2016a). Briefly, an inner aqueous phase of 500 μL was prepared with either water or 1 mg MOG_35__–__55_ in 500 μL water. For MPs with Enc excipients, mannitol, sucrose, or trehalose was loaded in the inner aqueous phase at 1%/5%/10% w/v. An organic phase of 80 mg of PLGA was dissolved in 5 mL dichloromethane; for Rapa-loaded MPs, 2 mg Rapa was loaded into the organic phase prior to synthesis. The inner aqueous and organic phases were sonicated for 30 s at 12 W to form a water/oil emulsion. The primary emulsion was then homogenized with an outer aqueous phase of 40 mL 2% w/v PVA for 3 min at 16,000 rpm. MPs were stirred at 350 rpm for 16 h to allow for solvent evaporation, then poured through a 40-μm cell strainer and collected using centrifugation (5 min at 5,000 *g*, 4°C). The supernatants were removed, and the MPs were washed three times in 1 mL water with collection by centrifugation after each wash. The particles were then resuspended in 1 mL of water. For MPs with Ext excipients, the MPs were resuspended in 1%/5%/10% w/v of mannitol, sucrose, or trehalose. For all synthesis cycles, a batch of control MPs was prepared without excipient. All of these control samples were analyzed in the cell culture studies to serve as batch validation. This resulted in a higher number of control samples (indicated in cell culture figures as “Lyophilized 0”) in this group than in the groups for the excipient formulations.

#### Lyophilization of MPs

Microparticles prepared with or without the cryoprotectants (mannitol, sucrose, or trehalose) incorporated either externally or by encapsulation at different concentrations (1, 5, and 10%) were placed into glass vials and flash frozen on dry ice. Frozen samples were loaded into a FreeZone 6-L Console −84°C Freeze Dryer (Labconco, Kansas City, MO, United States) and lyophilized over 48 h. Samples were removed from the freeze dryer, capped and sealed air-tight, and allowed at least 24 h to acclimate to room temperature and pressure before samples were characterized. Lyophilized samples were stored at room temperature.

### Characterization of Microparticles

#### Stability and Reconstitution Studies

Lyophilized MPs were stored at room temperature for 5 months post lyophilization. MPs were then reconstituted in 200 μL of water and vortexed for 10 s. To assess ease of reconstitution, supernatants were extracted, and remaining MPs were air dried. The mass of remaining MPs was measured. Percent reconstituted MP was calculated using the following equation:

$$\mathrm{Reconstitution}\mathrm{Efficiency}\left(\%\right)\;=\frac{\mathrm{Initial}\,\mathrm{Mass}\,\mathrm{MPs}-\mathrm{Remaining}\,\mathrm{Mass}\,\mathrm{MPs}}{\mathrm{Initial}\,\mathrm{Mass}\,\mathrm{MPs}}\times 100$$

#### MP Loading Analysis

To measure the loading of Rapa and MOG_35__–__55_ in MPs, a known volume of MPs was either air-dried or lyophilized to remove water from the sample. Dried MPs were dissolved in dimethyl sulfoxide. Rapa loading was measured by UV/Vis spectrophotometry, and MOG_35__–__55_ loading was measured using a Micro Bicinchoninic Acid (mBCA) Protein Assay kit (Thermo Fisher Scientific Pierce, Waltham, MA United States) according to the manufacturer’s instructions. Standard curves of known concentrations were used to calculate loading, reported as the mass of cargo per mass of dried MP formulation. Encapsulation efficiency was calculated by normalizing loading to drug input during MP synthesis:

$$\mathrm{Loading}\mathrm{Efficiency}\left(\%\right)=\frac{\mathrm{Drug}\,\mathrm{entrapped}\,\mathrm{in}\,\mathrm{MPs}}{\mathrm{Total}\,\mathrm{drug}\,\mathrm{added}\,\mathrm{initially}}\times 100$$

#### MP Size and Polydispersity Index

Particle size and polydispersity index (PDI) were measured using an LA-950 laser diffraction size analyzer (Horiba Instruments, Irvine, CA, United States). PDI was assessed using the following equation:

$$\text{PDI}={\left(\frac{\mathrm{standard}\,\mathrm{deviation}}{\mathrm{mean}\,\mathrm{diameter}}\right)}^{2}$$

### *In vitro* Cargo Functionality Cellular Assays

#### Cell Culture

Primary dendritic cells (DCs) were isolated from spleens of female C57BL/6J mice (The Jackson Laboratory, Bar Harbor, ME, United States, IMSR Cat# JAX:000664, RRID:IMSR_JAX:000664) female mice. DCs were isolated by positive selection for CD11c using a magnetic isolation kit according to the manufacturer’s instructions (Miltenyi Biotech, Bergisch Gladbach, Germany). Primary CD4^+^ MOG_35__–__55_ specific T cells were isolated from the spleens of transgenic “2D2” mice (C57BL/6-Tg(Tcra2D2,Tcrb2D2)1Kuch/J), (The Jackson Laboratory, IMSR Cat# JAX:006912, RRID:IMSR_JAX:006912). MOG_35__–__55_-specific T cells were isolated using a magnetic CD4^+^ T cell negative selection kit according to the manufacturer’s instructions (STEMCELL Technologies, Vancouver, BC, Canada).

All cells were cultured with DC 2.4 Media, comprised of RPMI1640 media + L-glutamine (Thermo Fisher Scientific, Gibco), supplemented with 10% fetal bovine serum (Sigma Aldrich), 1X Pen/Strep, 2 mM L-glutamine (Thermo Fisher Scientific, Gibco), 1X non-essential amino acids and 10 mM HEPES (Thermo Fisher Scientific, Hyclone GE Healthcare Life Sciences), and 55 μM β-mercaptoethanol (Sigma Aldrich), and incubated at 37°C.

All animal studies were fully compliant with local, state, and federal guidelines per the Association for Assessment and Accreditation of Laboratory Animal Care (AAALAC) expectations for animal care and use/ethics. All studies were approved and carried out under the supervision of the University of Maryland Institutional Animal Care and Use Committee (IACUC).

#### Flow Cytometry Studies

Following incubation times listed below, cells were prepared for analysis by flow cytometry. All antibodies were purchased from BD Biosciences (Franklin Lakes, NJ, United States). All analyses were completed performed on a FACS Celesta (BD Biosciences), and data analysis was conducted using FlowJo (BD Biosciences Tree Star, FlowJo, RRID:SCR_008520 version 10.6).

#### *In vitro* DC Activation Studies

Lyophilized MPs were stored at room temperature for 5 months. Fresh MPs for comparison were prepared without sugars the night before uptake studies. Primary DCs were seeded into a 96-well plate at 10^5^ cells per well and cultured with DC2.4 media. DCs were activated with lipopolysaccharide (Sigma Aldrich) at 0.25 μg/mL and then treated with 5–21 μg of MP formulation, dose-matched to provide 91 ng Rapa per well. Empty MPs were used as controls for Rapa-loaded MPs to assess differences due to MP formulation. Cells and MPs were cultured for 18 h. DCs were then stained for activation. Cells were first blocked with anti-CD16/CD32, then stained for CD11c, CD40 (BD Biosciences Cat# 553791, RRID:AB_395055), CD80 (BD Biosciences Cat# 560016, RRID:AB_1645212), CD86 (BD Biosciences Cat# 560582, RRID:AB_1727518), and viability using DAPI. Functionality of Rapa was assessed by a change in expression of DC activation markers. All flow cytometry data analysis was completed using Flowjo software. All samples were compared to Rapa MPs lyophilized with no excipient using Welch’s ANOVA with a Dunnett’s test for multiple comparisons.

#### *In vitro* T Cell Proliferation Studies

Lyophilized MPs were stored at room temperature for 5 months. Fresh MPs for comparison were prepared without sugars the night before uptake studies. Similar to DC activation studies, primary DCs were seeded into a 96-well plate at 10^5^ cells per well and cultured with DC2.4 media. DCs were activated with lipopolysaccharide at 0.25 μg/well and then treated with 5–10 μg of MPs, dose-matched to provide 68 ng of MOG_35__–__55_ per well. DCs and MPs were cultured for 24 h, after which MOG_35__–__55_-specific CD4^+^ T cells were added to the wells at 3 × 10^5^ cells per well. To assess T cell proliferation, prior to addition to the wells, T cells were labeled with Cell Proliferation Dye eFluor 450 (Thermo Fisher Scientific eBioscience). A media only and a soluble MOG_35__–__55_ + LPS served well as controls for low and high T cell proliferation, respectively. Cells were cultured for 72 h more and then stained for proliferation. Cells were first stained for viability using Fixable Viability Stain 510 (BD Biosciences), then blocked with anti-CD16/CD32 and stained for CD4 BD Biosciences (Cat# 552051, RRID:AB_394331). Functionality of MOG_35__–__55_ was evident by proliferation of MOG_35__–__55_-specific T cells treated with MPs loaded with MOG_35__–__55_. Rapa functionality was evident by a decrease in proliferation of T cells treated with MPs co-loaded with Rapa. All flow cytometry data analysis was completed using Flowjo software. The Proliferation Index of the sample was calculated using Flowjo Proliferation software analysis and is calculated using the following equation:

$$\mathrm{Proliferation}\,\mathrm{Index}=\frac{\mathrm{Total}\,\mathrm{Number}\,\mathrm{of}\,\mathrm{Cell}\,\mathrm{Divisions}}{\mathrm{Total}\,\mathrm{Number}\,\mathrm{of}\,\mathrm{Dividing}\,\mathrm{Cells}}$$

All samples were compared to MOG MPs lyophilized with no excipient using Welch’s ANOVA with a Dunnett’s test for multiple comparisons.

#### Cytokine Secretion

Inflammatory cytokine secretion was quantified by ELISA according to the manufacturer’s instructions (IFN-γ and IL-6, BD Biosciences; IL-17, Biotechne, R&D Systems). Media samples were collected from DC activation and T cell proliferation studies and analyzed for inflammatory cytokine concentrations. DC supernatants were run at 4× dilutions for IL-6. All IL-6 samples were compared to Rapa MPs lyophilized with no excipient using Welch’s ANOVA with a Dunnett’s test for multiple comparisons. T cell supernatants were run at 10× dilutions for IFN-γ and 5× dilutions for IL-17. IL-17 and IFN-γ samples were compared to MOG MPs lyophilized with no excipient using Welch’s ANOVA with a Dunnett’s test for multiple comparisons.

## Results

### Characterization of Microparticles

#### Incorporating Excipients Prior to Lyophilization Improves Lyo Cake Formation and Product Reconstitution

**FIGURE 1:**
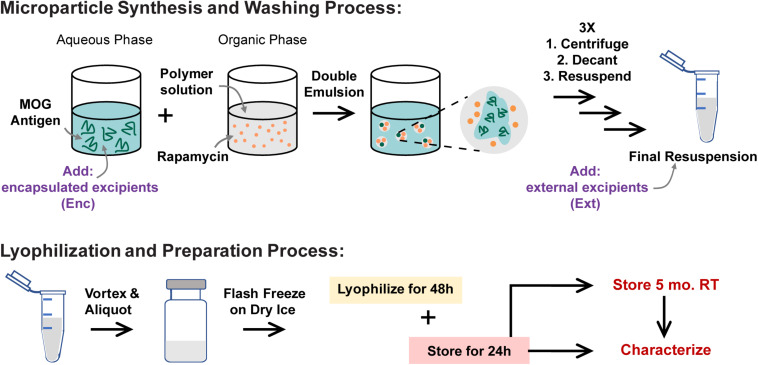
MP synthesis and lyophilization process. MPs are synthesized using a double-emulsion synthesis with an inner aqueous phase, organic phase, and outer aqueous phase mixed using sonication (first emulsion) and homogenization (second emulsion). The inner aqueous phase is loaded with water-soluble cargo including MOG peptide and any excipients that were to be encapsulated in the MPs (Enc). The organic phase contains the PLGA polymer and the water-insoluble cargo including Rapa. The outer aqueous phase contains 2% polyvinyl alcohol as a stabilizer. These MPs are washed and resuspended three times, after which any excipients that were to be externally incorporated are added (Ext). These MPs are mixed well and aliquoted into lyo vials, then flash frozen on dry ice. The frozen vials were lyophilized for 48 h, and then the lyophilized vials were allowed 24 h to acclimate to room temperature (RT) and atmospheric pressure before MPs were characterized for loading and sizing. MPs were stored for 5 months, then characterized and used in *in vitro* biofunctionality and reconstitution assays.

To determine if excipients improve collection and recovery of MP products, MPs were lyophilized with or without excipients. Excipients were either loaded into the MPs in the inner aqueous phase during synthesis (encapsulated—“Enc,” top left, Figure 1) or added after the final washing steps (externally incorporated—“Ext,” top right, Figure 1). Three excipients were selected based on previous research on protein stabilization in PLGA particles—mannitol, sucrose, and trehalose (Fonte et al., 2014, 2015, 2016a; Niu and Panyam, 2017). These were added to MP batches at concentrations of 1, 5, and 10% w/v during synthesis or after washing (Table 1). Samples were then lyophilized and stored (Figure 1, bottom).

**TABLE 1:** MOG/Rapa MP formulation information and abbreviations.

MP formulation	Cargo included	Excipient included	Excipient inclusion method	Concentration of excipient (%)
MR 0	MOG/Rapa	None	N/A	0
MR Man Enc 1	MOG/Rapa	Mannitol	Encapsulated	1
MR Man Enc 5	MOG/Rapa	Mannitol	Encapsulated	5
MR Man Enc 10	MOG/Rapa	Mannitol	Encapsulated	10
MR Man Ext 1	MOG/Rapa	Mannitol	External	1
MR Man Ext 5	MOG/Rapa	Mannitol	External	5
MR Man Ext 10	MOG/Rapa	Mannitol	External	10
MR Suc Enc 1	MOG/Rapa	Sucrose	Encapsulated	1
MR Suc Enc 5	MOG/Rapa	Sucrose	Encapsulated	5
MR Suc Enc 10	MOG/Rapa	Sucrose	Encapsulated	10
MR Suc Ext 1	MOG/Rapa	Sucrose	External	1
MR Suc Ext 5	MOG/Rapa	Sucrose	External	5
MR Suc Ext 10	MOG/Rapa	Sucrose	External	10
MR Tre Enc 1	MOG/Rapa	Trehalose	Encapsulated	1
MR Tre Enc 5	MOG/Rapa	Trehalose	Encapsulated	5
MR Tre Enc 10	MOG/Rapa	Trehalose	Encapsulated	10
MR Tre Ext 1	MOG/Rapa	Trehalose	External	1
MR Tre Ext 5	MOG/Rapa	Trehalose	External	5
MR Tre Ext 10	MOG/Rapa	Trehalose	External	10

*MR, MOG_35–55_/Rapa; Man, mannitol; Suc, sucrose; Tre, trehalose; Enc, encapsulated; Ext, externally incorporated; 0/1/5/10, concentration of excipient (w/v) in solution during addition step.*

**FIGURE 2:**
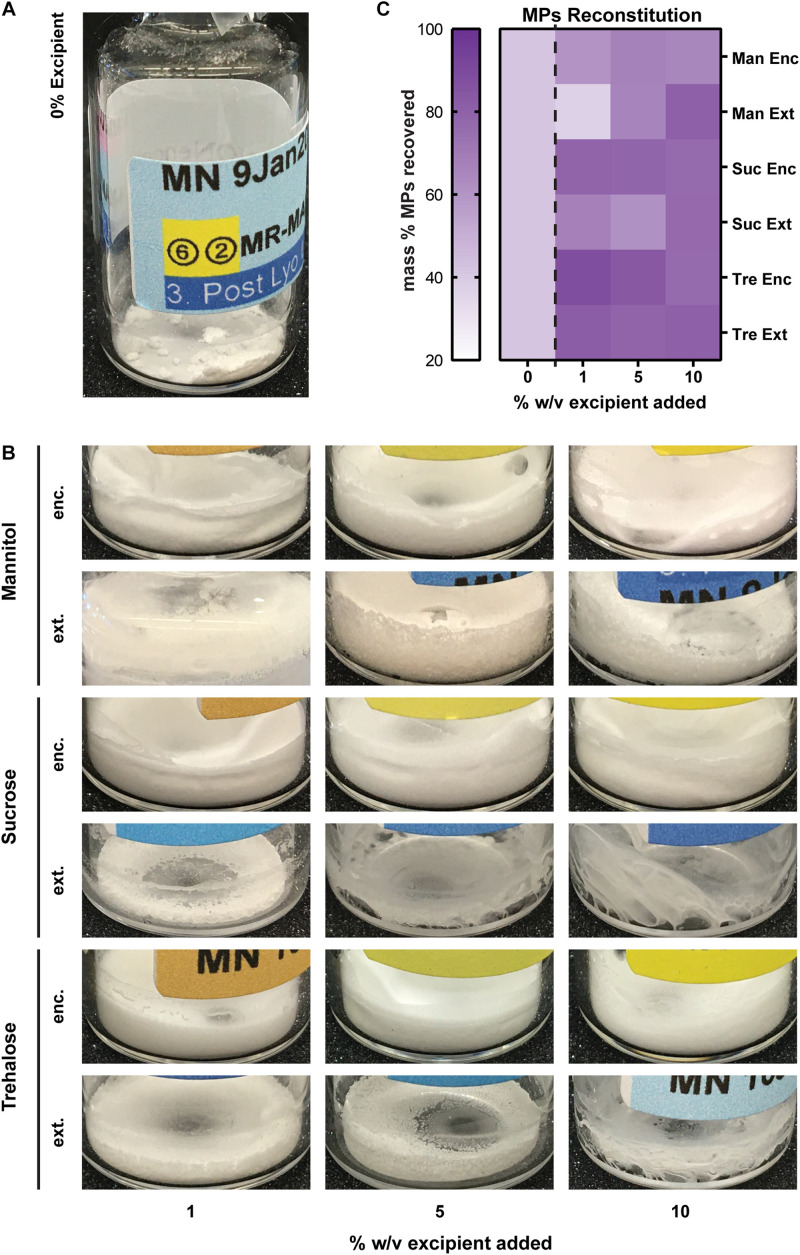
Caking, reconstitution, and product recovery as a function of excipient formulation. **(A)** MPs lyophilized and stored without excipients form fragile lyo cakes that crumble apart. **(B)** Images of MPs with encapsulated excipients. **(C)** Mass recovered of each formulation following a reconstitution assay (solution addition and vortexing for 10 s).

To assess the quality of the cakes formed by MPs after lyophilization, the lyo cakes were imaged to reveal the extent of crystallization and cake formation. High-quality lyo cakes appear smooth with even color and do not contain crevices or pockets from ice crystal formation. These data revealed that MPs lyophilized and stored without excipients formed fragile lyo cakes that easily crumble (Figure 2A). Qualitatively, MPs with Enc excipients generally formed lyo cakes that appeared less brittle than MPs without excipients (Figure 2B). When comparing within the excipient formulations, there were some difference, but no trends were evident across the excipients. This generally suggests that the primary driver of cake formation is the presence of excipient, with relative insensitivity to the specific excipient and concentration. To quantitatively determine the ease of reconstitution of the MP lyophilization products, water was added to each sample, then vortexed for 10 seconds. The solutions were collected and used to determine the extent of mass recovery. MPs with excipient were more easily reconstituted relative to those without excipients, as indicated by remaining material in the vials after reconstitution solutions were removed (Supplementary Figure 1). In particular, for nearly all cases, this corresponded to an increase in MP mass recovery in the reconstitution solutions (Figure 2C and Supplementary Table 1). Across the excipient concentrations and stage of excipient addition (i.e., Enc, Ext), trehalose conferred the most consistent improvements in recovery, followed by sucrose and mannitol. The only formulation that did not improve recovery relative to cakes without excipient was the lowest concentration of mannitol added externally (Figure 2C).

#### Cargo Loading Efficiency Is Influenced by Excipient Formulation

**FIGURE 3:**
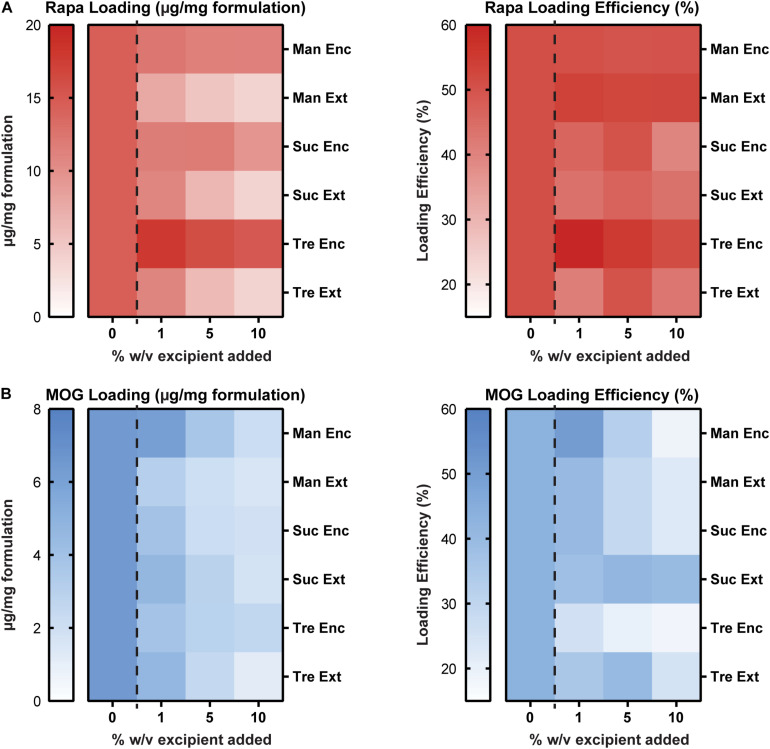
Cargo loading levels and loading efficiency as a function of excipient formulation. **(A)** Rapa loading per mg of formulation **(left)** and Rapa loading efficiency relative to initial drug input **(right)**. **(B)** MOG loading per mg of formulation **(left)** and MOG loading efficiency relative to initial MOG input **(right)**.

We next studied the impact of excipients on immune cargo loading using UV/Vis spectrophotometry and mBCA assays to assess Rapa and MOG loading (μg/mg), respectively. In these studies, Enc excipient maintained and improved Rapa loading compared to externally added (Ext) excipient (Figure 3A, left and Supplementary Table 2). The loading levels for Enc Rapa were similar to MPs without excipient, though Enc trehalose increased Rapa loading. Within the Ext excipient formulations, there was an inverse concentration effect, which is expected as the mass of excipient increases with increasing excipient concentration. The efficiency of loading was a weaker function of the excipient formulations, though a maximum was observed when a low concentration (i.e., 1%) of Enc trehalose was used (Figure 3A, right and Supplementary Table 2). Reduced values were observed for both sucrose addition schemes and the externally added trehalose, with some modest concentration effects. MOG loading decreased as excipient concentration increased (Figure 3B, left and Supplementary Table 2). However, no clear trends emerged as a function of the specific sugar structure or addition scheme. A generally similar inverse concentration trend was observed for the loading efficiency, but the reduction was not present for sucrose and trehalose added externally; in the latter case, the highest efficiency was measured for the intermediate excipient concentration (Figure 3B, right and Supplementary Table 2). In a few cases, the data in Figure 3 reveal decreases in loading levels, while loading efficiency increases. The loading level is driven by the cargo loading per mass of formulation and the efficiency is determined in part by the mass of formulation recovered, which is impacted by the addition of excipient (Figure 2). Taken together, these data suggest that excipients impact not only particle recovery but also the cargo loading levels and efficiency. Further, these data suggest that at high concentrations of excipients, there is a possibility for MOG cargo to be displaced. Thus, in considering translation and therapeutic use, a related variable that would be important to study with respect to lyophilization and stabilization is the ratio of MOG and Rapa cargo in a given candidate formulation.

#### Effect of Excipient Formulation on MP Size and Size Distribution

**FIGURE 4:**
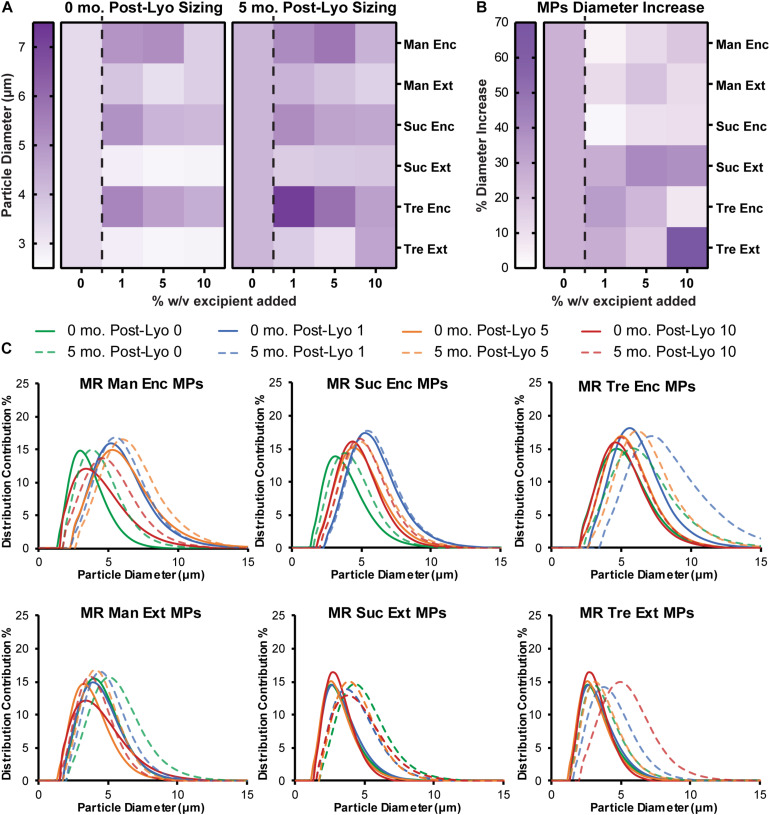
Encapsulated excipients increase MP size, and adding excipients protects against aggregation. **(A)** MPs were sized immediately following lyophilization and again after storage at room temperature for 5 months. **(B)** MP size data before and after storage was used to report a percent increase in diameter. **(C)** Size curves for each excipient formulation before and after storage for 5 months.

To assess the stability and aggregation state of MPs formed with excipients, diameter and PDI were monitored. MPs with each excipient formulation were sized before lyophilization (Supplementary Figure 2), after lyophilization (Figure 4A, left), and after 5 months in storage (Figure 4A, right and Supplementary Table 3). Encapsulating excipients within the MPs generally increased the diameter of MPs, but this effect diminished as the concentration increased. In contrast, the externally added excipients minimized the diameter after lyophilization. Importantly, for sucrose and trehalose—where the effects were greatest—these diameters were smaller than the diameters of MPs lyophilized without excipient. After storage for 5 months and reconstitution, MPs generally increased in size (Figure 4A), with the largest increases observed in particles that exhibited the smallest diameters before the storage period (i.e., Suc Ext, Tre Ext) (Figure 4B). While these formulations exhibited larger increases in size, the particles that exhibited larger diameters immediately after lyophilization (i.e., Man Enc, Man Ext, Suc Enc) revealed smaller increases in size during the storage period (Figure 4B). These findings were also confirmed by analysis of particle size distribution (Figure 4C). Importantly, encapsulating (Enc) excipients into the MP formulations also reduced the PDI relative to MPs without excipients, a favorable outcome for product manufacturing and uniformity (Figure 4C and Supplementary Figure 3). Taken together, the data reveal that excipients can reduce particle diameter and PDI during lyophilization processes relative to MPs without excipients.

### *In vitro* Cargo Functionality Cellular Assays

#### Effect of Excipient Formulation on DC Viability and Deactivation After Stimulation

**FIGURE 5:**
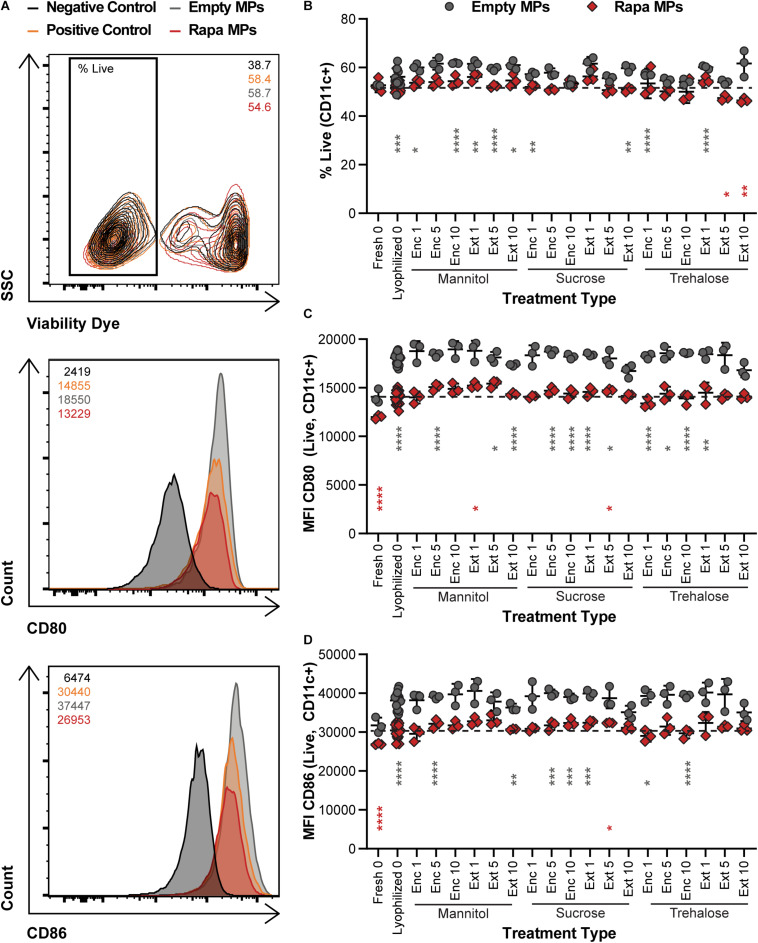
Effect of MP excipient formulation on DC viability and deactivation after stimulation. Primary DCs were collected and cultured for 18 h with LPS and empty or Rapa-loaded MPs to assess changes in viability and deactivation of stimulated DCs. **(A)** Flow cytometry gating scheme for DC activation analysis. The numbers in the top right of the contour plot indicate viability for the corresponding representative plot, while the numbers in the top left corner of the histograms indicate the median fluorescent intensity (MFI) for the corresponding representative plot. **(B)** Viability of DCs. Activation following treatment, as measured by CD80 **(C)** and CD86 **(D)**. Gray and red asterisks, respectively, indicate a specific empty MP (gray) or Rapa MP (red) formulation is significant against Rapa MPs without excipient (“Lyophilized 0”). For reference, the comparison values are indicated using dashed lines in each panel. For all panels, statistical comparisons were performed using Welch’s ANOVA with a Dunnett’s test for multiple comparisons. (*p ≤ 0.05, **p ≤ 0.01, ***p ≤ 0.001, ****p ≤ 0.0001). The legend in panel A applies to all gating schemes in panel **(A)**. The legend above panel **(B)** applies to panels **(B–D)**.

To confirm that the potency of immune cargo is retained after lyophilization with excipients, changes in DC activation were assessed following treatments with Rapa-loaded MPs. After isolation, DCs were treated with LPS, which induces an inflammatory response in DCs, and either Empty (X) MPs or Rapa-loaded (R) MPs. For these candidate therapies, the Rapa cargo plays a role in reducing the activation of DCs, an important step to restrain the downstream destructive effects of self-reactive T cells. The viability of the cells was then quantified, along with the deactivation as a result of Rapa treatment using the median fluorescence intensity (MFI) of common DC co-stimulatory signals, CD80 and CD86. Figure 5A shows the gating scheme with representative samples (negative control, positive control, an Empty MP sample, and a Rapa MP sample). Generally, viability was a weak function of treatment type, with all samples exhibiting typical viability levels for culture of primary DCs (Figure 5B and Supplementary Figure 4A). As expected, many of the empty MP formulations conferred a modest increase relative to Rapa MPs lyophilized without excipient (gray asterisks) because Rapa can restrain processes involved in cell division and maintenance. With respect to Rapa MP excipient formulations, Ext trehalose at the 5 and 10% excipient concentrations (red asterisks) resulted in modest decreases in viability, though the size of the decrease was small. In assessing deactivation of the LPS-stimulated DCs, the presence of Rapa in the MPs was the main driver of outcome. For both CD80 (Figure 5C) and CD86 (Figure 5D), MPs containing Rapa for almost all excipient formulations reduced activation to levels equivalent to those measured for freshly lyophilized MPs without excipient. These levels were also similar to those in controlled studies using soluble Rapa as a deactivation control (Supplementary Figures 4B,C). A few of the Rapa MP excipient formulations (red asterisks) caused variations relative to the freshly lyophilized Rapa MPs without excipient, but again, these shifts were modest in scale. Together, these data confirm that long-term storage of Rapa MPs does not impact the suppressive function of Rapa across a range of excipient designs.

#### MPs Containing MOG Maintain Antigen Integrity and Expand MOG-Specific T Cells After Long-Term Storage

**FIGURE 6:**
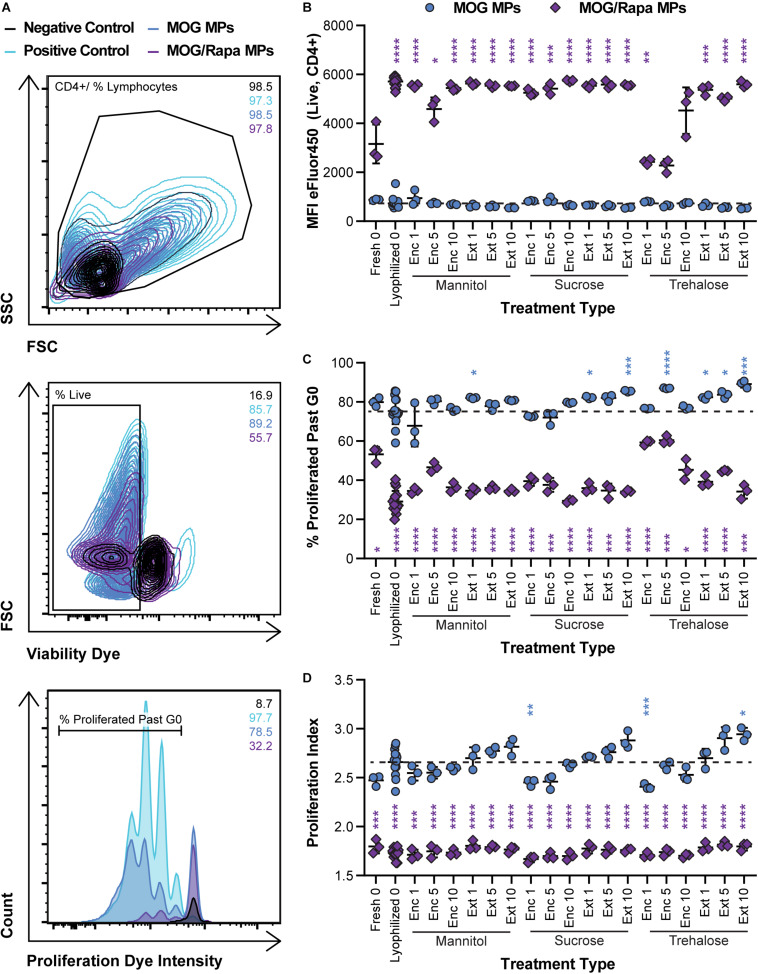
Effect of MP excipient formulation on expansion of MOG-specific T cells. Primary DCs were collected and cultured for 24 h with LPS and either MOG or MOG/Rapa MPs. After 24 h, fluorescently labeled MOG-specific transgenic T cells were added to wells and cultured for 72 h to assess proliferation of MOG-specific T cells. **(A)** Flow cytometry gating scheme for T cell proliferation analysis. **(B)** MFI of fluorescently labeled (eFluor450) MOG-specific T cells as an indicator of proliferation. **(C)** Frequency (%) of proliferated MOG-specific T cells. **(D)** Proliferation index, reflecting the average number of divisions undergone by proliferating T cells. Blue and purple asterisks, respectively, indicate a specific MOG MP (blue) or MOG/Rapa MP (purple) formulation is significant against MOG MPs lyophilized with no excipients (“Lyophilized 0”). For reference, the comparison values are indicated using dashed lines in each panel. For all panels, statistical comparisons were performed using Welch’s ANOVA with a Dunnett’s test for multiple comparisons. (*p ≤ 0.05, **p ≤ 0.01, ***p ≤ 0.001, ****p ≤ 0.0001). The legend in panel **(A)** applies to all gating schemes in panel **(A)**. The legend in above panel **(B)** applies to panels **(B–D)**.

We next determined if lyophilization and storage impacts the ability of MPs to limit proliferation of MOG-specific T cells. MPs loaded with MOG or MOG/Rapa were synthesized and stored with or without excipients. These formulations were then cocultured with DCs and fluorescently labeled T cells isolated from 2D2 transgenic mice. T cells from this strain exhibit receptors specific for MOG. Thus, when these cells encounter MOG displayed by DCs with appropriate co-stimulatory signals, they proliferate and secrete inflammatory cues. Proliferation was assessed by quantifying the intensity of dye in the T cells at the end of culture, where a decrease in signal corresponds to an increase in proliferation as a result of dye dilution during successive generations of cell division. Figure 6A shows the gating scheme with representative samples (negative control, positive control, a MOG MP sample, and a MOG/Rapa MP sample). As expected, all MOG MP excipient formulations caused strong T cell expansion, as indicated by the low MFI values (i.e., dilution of proliferation dye) (Figure 6B and Supplementary Figure 5A). In the case of MOG/Rapa MPs, most formulations potently suppressed proliferation, with a few exceptions (e.g., trehalose “Enc 1%,” “Enc 5%”). While there were no clear indicators why this occurred, further study could reveal effects from excipient concentration or variation in relative cargo loading when multiple components are present. We also observed that these formulations exhibited the largest sizes and widest size distributions, shown in Figure 4, after storage. Together, the data on these formulations might indicate that the large size increases or aggregation limited the ability of cargo to be internalized or presented, or altered the kinetics of these processes to drive proliferation.

Assessing the frequency (%) of cells that proliferated past generation 0 (G0) revealed similar trends (Figure 6C and Supplementary Figure 5B) but allowed visualization of some subtler differences within the MOG MP groups. For both sucrose and trehalose, several MOG MP formulations increased proliferation frequency relative to MOG MPs lyophilized without excipient. However, there were no clear trends with respect to the excipient concentration or stage of addition. To directly assess the number of divisions of each T cell, we next calculated proliferation indices (Figure 6D and Supplementary Figure 5C). All of the MOG/Rapa MP excipient formulations significantly decreased the proliferation index relative to MOG MPs lyophilized without excipient, but there were no differences as a function of the specific excipient schemes. For the MOG MPs, the majority of the samples were not statistically different from the MOG MPs lyophilized without excipient. For each excipient, there was a trend of increased proliferation moving from Enc to Ext, and also as a function of concentration. However, these were only significant in the case of Ext at the highest concentration. Overall, the data Figures 5, 6 indicate that—across a range of excipient formulations—MOG/Rapa MPs maintain their ability to deactivate DCs and restrict myelin-reactive T cells following lyophilization and long-term storage.

#### MPs Containing MOG/Rapa With or Without Excipients Can Reduce IL-6, IL-17, and IFN-γ Inflammatory Cytokine Secretion

**FIGURE 7:**
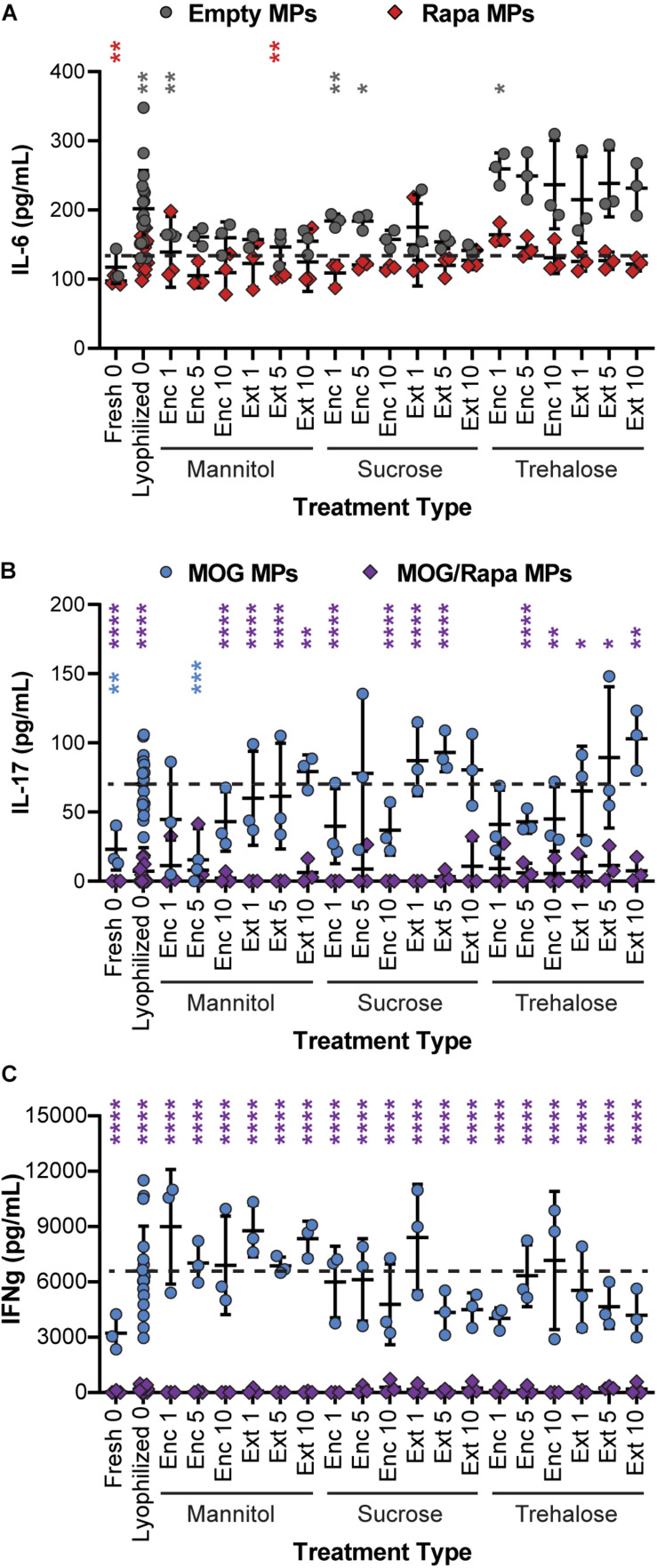
Effect of excipient formulation on inflammatory activity of immune cells. Functional analysis of immune cells in *in vitro* cultures assessed by ELISAs for common inflammatory cytokines, **(A)** IL-6 secretion by DCs, **(B)** IL-17, and **(C)** IFN-γ secretion by T cells. Samples in panel A were compared to Rapa MPs lyophilized with no excipient using Welch’s ANOVA with a Dunnett’s test for multiple comparisons. Samples in panels B and C were compared to MOG MPs lyophilized with no excipient using Welch’s ANOVA with a Dunnett’s test for multiple comparisons. (*p ≤ 0.05, **p ≤ 0.01, ***p ≤ 0.001, ****p ≤ 0.0001). The color of an asterisk indicates a specific formulation is significant against Rapa MPs (“Lyophilized 0”) in panel **(A)**, or MOG MPs (“Lyophilized 0”) in panels **(B,C)**. For reference, the comparison values are indicated using dashed lines in each panel. The legend above panel **(B)** applies to panels **(B,C)**.

To determine if excipient formulation impacts not only T cell proliferation but also the ability of cargo to exert the desired anti-inflammatory effects, supernatants were collected from the DC/T cell cocultures and analyzed for cytokine secretion using ELISA. In particular, we assessed IL-6 secreted by DCs, along with IFN-γ and IL-17 secreted by T cells. IL-6 promotes acute phrase proteins and lymphocyte proliferation, IFN-γ promotes cellular immunity and control of intracellular pathogens, and IL-17 is secreted by T_H_17 effector cells that support further inflammatory cytokine secretion and the role of monocytes and neutrophils. IL-6 secretion was a weak function of excipient formulation, with many samples—both Empty MPs and Rapa MPs—exhibiting no statistical differences relative to the Rapa MPs lyophilized without excipient (Figure 7A and Supplementary Figure 6A). This suggests that the suppression of IL-6 secretion is not sensitive to the specific excipient chosen. In a few cases, Empty MPs resulted in modest increases in IL-6; this was most obvious in particles using trehalose as an excipient, though most of these were not statistically significant. This could suggest that trehalose exhibits some inherent immunogenic properties, though additional studies would be needed to confirm this possibility.

In assessing IL-17 (Figure 7B and Supplementary Figure 6B), we found that nearly all of the MOG/Rapa MPs significantly suppressed IL-17 production relative to MOG MPs lyophilized without excipients. These results were irrespective of the specific excipient formulation. There were a few exceptions for IL-17, but no consistent trends were obvious. In line with the data on proliferation index, MOG MPs did exhibit a weak trend, with IL-17 secretion increasing with excipient concentration and when comparing Enc excipient vs. Ext excipient. Although therapeutic particles based on these MPs would likely contain both MOG and Rapa, this weak trend does suggest that MPs containing only the self-antigen might best be stabilized by encapsulating excipient (“Enc”) to minimize inflammatory cytokines. Lastly, for IFN-γ secretion (Figure 7C and Supplementary Figure 6C), all of the MOG/Rapa MPs restrained secretion relative to MOG MPs lyophilized without excipient. None of the MOG MP formulations with excipients suppressed IFN-γ relative to the MOG MPs without excipient. Taken together, these functional cytokine measurements confirm that a range of excipient strategies can be used to stabilize the particles, without impacting the immunological function of these cargos. Thus, there is opportunity to select excipient types, concentrations, and stages of addition that are favorable for manufacturing and recovery while maintaining a target product profile.

## Discussion

As immunotherapies become more personalized and patient-specific to improve patient outcomes, biomaterials are becoming increasingly important as delivery agents (Andorko et al., 2015; Northrup et al., 2016; Tostanoski et al., 2016b; Bookstaver et al., 2018; Ben-Akiva et al., 2019). Biomaterials provide incredible flexibility in stabilizing and delivering immunotherapy cargo, as they are highly tunable, with properties that can be engineered to meet many different functions (Fang and Zhang, 2016; Gammon et al., 2016; Tostanoski et al., 2016b). Importantly, they can provide features that traditional immune modulators do not have—including co-delivery of immune signals, controlled release of immune cues, and prolonged immune cargo exposure and protection (Tostanoski et al., 2016b; Bookstaver et al., 2018). However, long-term storage of biomaterial-based immunotherapies has different considerations than long-term storage of other biologics used in immunotherapies (e.g., monoclonal antibodies), due to those specific functions, which adds complexity for manufacturing and regulatory characterization. As preclinical studies often allow for the production of immunotherapies on-demand, relatively few preclinical studies have been focused on improving storage and stability of biomaterial-based immunotherapies. However, in order to translate these immunotherapies to the clinic, more research is required in these areas.

For our studies, we used a well-established material—PLGA—to deliver our immunotherapy cargo—a myelin peptide and immunomodulatory signal rapamycin. PLGA is commonly used in applications for humans, as it degrades by hydrolysis, and its degradation products can be processed by cells (Danhier et al., 2012; Mohammadi-Samani and Taghipour, 2015). However, it is this same hydrolysis degradation process that complicates storage of immunotherapies based on PLGA MPs, as aqueous storage of PLGA MPs can lead to undesirable cargo release, particle aggregation and fusion, and degradation of cargo integrity (Fonte et al., 2016b). Thus, we turned to lyophilization—a common method used in the pharmaceutical industry to control dehydration of samples—to enhance the stability of PLGA MPs during storage. As the MP structure can be damaged from the stress of lyophilization due to ice crystal formation (Holzer et al., 2009; Fonte et al., 2016b), we tested several lyoprotectants to protect against ice crystal formation and immobilize MPs to limit aggregation. We tested several features to compare MPs lyophilized and stored for 5 months without excipients to MPs lyophilized and stored for 5 months with excipients, and against MPs made on-demand, fresh immediately before use.

Throughout our materials and cellular testing, several findings emerged. First, lyophilizing MPs with excipients—both Enc and external—improved yield of lyophilization cakes. Observationally, these cakes also appeared to be more stable and less broken relative to MPs lyophilized without excipient. These considerations are important, as damaged lyophilized formulations can possess unfavorable properties, for example, component crystallization and higher residual water content (Izutsu et al., 2014). Further studies, such as x-ray diffraction or differential scanning calorimetry, would be useful in investigating the crystallization state and water content as a function of lyophilization formulation. Qualitatively, in our studies, samples lyophilized with excipients Enc exhibited the smoothest appearances, with the fewest visible pores in the bulk sample. Improved lyo cake formation with lower concentrations of Ext excipients is not unique to PLGA MPs. Protein preservation efforts in immunotherapy, for example, have concluded that lowering concentrations of Trehalose excipient reduces formation of a glassy matrix and leads to more concentrations of proteins during freezing (Hassett et al., 2013). We also measured improvements in ease of reconstituting MPs formulated with excipients. With the exception of the 1% Enc mannitol formulation, all MPs with excipients showed more complete reconstitution after a 10-second vortex. This is important: in an environment where immunotherapies are carefully titrated and tested prior to approval, it is essential that patients get the entire dose.

Since dosing is such an important consideration, effective co-loading of immune cargo is another feature of PLGA MPs that must be supported by the preparation process. MOG and Rapa can both be loaded into PLGA MPs without excipients relatively efficiently (43.5 and 51.0%, respectively). In polymer-based drug carrier systems, studies have suggested that excipients like sucrose may induce conformation changes in protein cargo (Vlugt-Wensink et al., 2007). Increasing hydrophobicity of a protein could increase its partitioning over the organic phase and reduce its concentration and therefore loading within the inner aqueous core (Vlugt-Wensink et al., 2007). This suggests that lower excipient inclusion levels (1%) might be the best for this application. This aligns well with the results from the lyo cake formation and reconstitution assay. However, studies focused on minimizing variability of relative cargo loading for a range of input cargos would increase the robustness of this approach.

Our lab has previously used these MPs to deliver MOG and Rapa to lymph nodes to reprogram autoimmune responses to myelin (Tostanoski et al., 2016a). We directly inject MPs into lymph nodes (Andorko et al., 2014) and rely on the size of the PLGA MPs (∼2–5 μm) to prevent drainage from lymph nodes and uptake by non-phagocytic cells (Singh et al., 2007; Jewell et al., 2011; Keselowsky et al., 2011; Andorko et al., 2016; Tostanoski et al., 2016a; Gammon et al., 2017; Gosselin et al., 2017). However, APC uptake is much less efficient once MPs reach sizes >5 μm (Singh et al., 2007). After synthesis and lyophilization, MOG/Rapa MPs without excipients are on average 3.4 μm in diameter, with all of the MPs with Ext excipients in the same range (2.7–3.8 μm). Adding Enc excipients to the aqueous phase of PLGA MPs increases the mean diameter of the batch to sizes after lyophilization to about 4.5 μm (3.6–5.4 μm). However, after storage for 5 months, several MOG/Rapa PLGA MPs without excipients experience greater increases in size and size distribution compared to the MPs stored with excipients. In particular, the MPs prepared with mannitol (both enc and ext), as well as the sucrose and trehalose enc (with the exception of 1% tre enc) MPs, best maintained their size after storage.

In order to maintain the benefits of a biomaterial-based immunotherapy, the excipients added to enhance storage must enable biofunctionality of immune cargo and not interrupt with drug–target interaction. During DC culture studies, empty MPs—before and after lyophilization and storage—exhibited higher levels of activation relative to Rapa MPs. This was also seen in several cases when measuring IL-6 secretion. This may result from potentially immunogenic features of the polymer or polymer degradation—PLGA is known to have some intrinsic immunogenicity as it degrades (Sharp et al., 2009; Park and Babensee, 2012; Park et al., 2015). However, in all cases, adding rapamycin to these particles, with or without excipients, decreased the expression of DC activation markers, showing that the Rapa maintains its immunosuppressive activity during storage with or without excipients. The MOG-specific T-cell coculture revealed lyophilized MOG MPs—with or without excipients—can stimulate T-cell proliferation comparably to fresh MOG MPs, confirming that the MOG in the MPs maintains biofunctionality. In addition, adding Rapa to these MPs often significantly restrains this proliferation compared to lyophilized MOG MPs without excipients. In fact, MOG/Rapa MPs lyophilized with or without excipients restrain proliferation similarly to fresh MOG/Rapa MPs. These trends are also seen in the inflammatory IL-17 and IFN-γ, suggesting that the intrinsic immunogenicity of the slightly degrading PLGA is not enough to impair the biofunctionality of the loaded immune cargo.

Effects of excipients on cellular viability and function can greatly impact the success of a vaccine carrying out proper function (Kheddo et al., 2016). The excipients assisted in preserving desirable properties of the particles without substantial impacts on the immunologic and cell profiles. However, our list of candidate lyoprotectants is not exhaustive. Classes of molecules other than sugars have been studied as excipients for lyophilization. Arginine and glutamate, for example, have been of interest in immunotherapy design due to beneficial function that the amino acids have as excipients (Blobel et al., 2011; Kheddo et al., 2016). Generally, amino acids are natural constituents of cells and are not a threat to cellular viability. Studies of these residues have also shown increased protein stability with vaccine development (Blobel et al., 2011).

While, in this study, a full optimization of the lyophilization process itself was not the focus, controlling these parameters can further optimize preservation of the candidate therapeutics. One important parameter is the shelf temperature (Izutsu et al., 2014). Controlling the chamber pressure can provide further fine tuning with an aim of ice sublimating as rapidly as possible so as not to cause any physical changes in the solid phase of the product. Other strategies such as post-freeze heat treatment, ultrasound ice fog, vacuum-induced surface freezing, and release of the pressurized/depressurized chamber are also possible to preserve biologics and their carriers (Izutsu et al., 2014). Cryopreservation is a simple and available technique that can extend the usefulness of drug carriers (Izutsu et al., 2014; Fonte et al., 2015, 2016b). Implementing these strategies would benefit MP stabilization. The results obtained from our studies and further exploration that is evident in the field of vaccine design presents promise in development of a preservation method for biomaterial-based immunotherapies.

## Conclusion

Understanding and optimizing the stability of biomaterials-based immunotherapies will enable translation of these immunotherapies beyond preclinical studies. In this study, we incorporated excipients in the lyophilization process of PLGA MPs loaded with peptide self-antigens and small molecule immunomodulators to enhance the stability of cargo-loaded MPs. The presence of the excipients during the lyophilization process improving lyo cake formation and conferred favorable reconstitution properties and MP recovery yields. These benefits were achieved without impacting the function of peptide and small-molecule cargos used to restrain immune response in primary cells. Utilizing these agents in biomaterial systems aimed at tolerance provides several levers to control important post-synthesis processes.

## Data Availability Statement

The original contributions presented in the study are included in the article/Supplementary Material, further inquiries can be directed to the corresponding author/s.

## Ethics Statement

All animal studies were fully compliant with local, state, and federal guidelines per the Association for Assessment and Accreditation of Laboratory Animal Care (AAALAC) expectations for animal care and use/ethics. All studies were carried out under the supervision of the University of Maryland Institutional Animal Care and Use Committee (IACUC).

## Author Contributions

MN, EG, and CJ designed the experiments and wrote the manuscript. MN, EG, and SB performed all experiments. MN and EG prepared and characterized all MP formulations. EG performed all *in vitro* cell studies. SB contributed to characterization studies and *in vitro* cell studies. All authors contributed to data analysis and manuscript preparation.

## Disclaimer

The views reported in this paper do not reflect the views of the Department of Veterans Affairs or the United States Government.

## Conflict of Interest

CJ is an employee of the VA Maryland Health Care System. CJ has equity positions with Avidea Technologies and Cellth Systems, LLC. The remaining authors declare that the research was conducted in the absence of any commercial or financial relationships that could be construed as a potential conflict of interest.

## Abbreviations

X: empty microparticles
R: Rapa microparticles
M = MOG: microparticles
MR = MOG/Rapa: microparticles
Pre-Lyo: pre lyophilization
Post-Lyo: post lyophilization
5 mo. Post-Lyo: 5 months Post lyophilization
Man: mannitol
Suc: sucrose
Tre: trehalose
Enc: encapsulated
Ext: externally loaded.

## References

[B1] Andorko J. I. Gammon J. M. Tostanoski L. H. Zeng Q. Jewell C. M. (2016). Targeted programming of the lymph node environment causes evolution of local and systemic immunity. *Cell. Mol. Bioeng.* 9 418–432. 10.1007/s12195-016-0455-6 27547269 PMC4978773

[B2] Andorko J. I. Hess K. L. Jewell C. M. (2015). Harnessing biomaterials to engineer the lymph node microenvironment for immunity or tolerance. *AAPS J.* 17 323–338. 10.1208/s12248-014-9708-2 25533221 PMC4365095

[B3] Andorko J. I. Tostanoski L. H. Solano E. Mukhamedova M. Jewell C. M. (2014). Intra-lymph node injection of biodegradable polymer particles. *J. J. Vis. Exp.* 83:6. 10.3791/50984 24430972 PMC4047663

[B4] Ben-Akiva E. Witte S. E. Meyer R. A. Rhodes K. R. Green J. J. (2019). Polymeric micro- and nanoparticles for immune modulation. *Biomater. Sci.* 7 14–30. 10.1039/c8bm01285g 30418444 PMC6664797

[B5] Bitsch A. Schuchardt J. Bunkowski S. Kuhlmann T. Bruck W. (2000). Acute axonal injury in multiple sclerosis - Correlation with demyelination and inflammation. *Brain* 123 1174–1183. 10.1093/brain/123.6.1174 10825356

[B6] Blobel J. Brath U. Bernadó P. Diehl C. Ballester L. Sornosa A. (2011). Protein loop compaction and the origin of the effect of arginine and glutamic acid mixtures on solubility, stability and transient oligomerization of proteins. *Eur. Biophys. J.* 40 1327–1338. 10.1007/s00249-011-0686-3 21390527

[B7] Bookstaver M. L. Tsai S. J. Bromberg J. S. Jewell C. M. (2018). Improving vaccine and immunotherapy design using biomaterials. *Trends Immunol.* 39 135–150. 10.1016/j.it.2017.10.002 29249461 PMC5914493

[B8] Chishti N. Jagwani S. Dhamecha D. Jalalpure S. Dehghan M. H. (2019). Preparation, optimization, and in vivo evaluation of nanoparticle-based formulation for pulmonary delivery of anticancer drug. *Medicina* 55:294. 10.3390/medicina55060294 31226865 PMC6631245

[B9] Comabella M. Khoury S. J. (2012). Immunopathogenesis of multiple sclerosis. *Clin. Immunol.* 142 2–8. 10.1016/j.clim.2011.03.004 21458377

[B10] Cross A. H. Naismith R. T. (2014). Established and novel disease-modifying treatments in multiple sclerosis. *J. Intern. Med.* 275 350–363. 10.1111/joim.12203 24444048

[B11] Danhier F. Ansorena E. Silva J. M. Coco R. Le Breton A. Preat V. (2012). PLGA-based nanoparticles: an overview of biomedical applications. *J. Control. Release* 161 505–522. 10.1016/j.jconrel.2012.01.043 22353619

[B12] Dellacherie M. O. Seo B. R. Mooney D. J. (2019). Macroscale biomaterials strategies for local immunomodulation. *Nat. Rev. Mater.* 4 379–397. 10.1038/s41578-019-0106-3

[B13] Fang R. N. H. Zhang L. F. (2016). “Nanoparticle-based modulation of the immune system,” in *Annual Review of Chemical and Biomolecular Engineering*, Vol. 7 ed. Prausnitz J. M. (Palo Alto: Annual Reviews), 305–326. 10.1146/annurev-chembioeng-080615-034446 27146556

[B14] Fenton O. S. Olafson K. N. Pillai P. S. Mitchell M. J. Langer R. (2018). Advances in biomaterials for drug delivery. *Adv. Mater.* 30:29. 10.1002/adma.201705328 29736981 PMC6261797

[B15] Fonte P. Andrade F. Azevedo C. Pinto J. Seabra V. van de Weert M. (2016a). Effect of the freezing step in the stability and bioactivity of protein-loaded PLGA nanoparticles upon lyophilization. *Pharm. Res.* 33 2777–2793. 10.1007/s11095-016-2004-3 27444681

[B16] Fonte P. Araújo F. Seabra V. Reis S. van de Weert M. Sarmento B. (2015). Co-encapsulation of lyoprotectants improves the stability of protein-loaded PLGA nanoparticles upon lyophilization. *Int. J. Pharm.* 496 850–862. 10.1016/j.ijpharm.2015.10.032 26474964

[B17] Fonte P. Reis S. Sarmento B. (2016b). Facts and evidences on the lyophilization of polymeric nanoparticles for drug delivery. *J. Control. Release* 225 75–86. 10.1016/j.jconrel.2016.01.034 26805517

[B18] Fonte P. Soares S. Sousa F. Costa A. Seabra V. Reis S. (2014). Stability study perspective of the effect of freeze-drying using cryoprotectants on the structure of insulin loaded into PLGA nanoparticles. *Biomacromolecules* 15 3753–3765. 10.1021/bm5010383 25180545

[B19] Gammon J. M. Dold N. M. Jewell C. M. (2016). Improving the clinical impact of biomaterials in cancer immunotherapy. *Oncotarget* 7 15421–15443. 10.18632/oncotarget.7304 26871948 PMC4941251

[B20] Gammon J. M. Gosselin E. A. Tostanoski L. H. Chiu Y.-C. Zeng X. Zeng Q. (2017). Low-dose controlled release of mTOR inhibitors maintains T cell plasticity and promotes central memory T cells. *J. Control. Release* 263 151–161. 10.1016/j.jconrel.2017.02.034 28257991 PMC5573661

[B21] Gosselin E. A. Eppler H. B. Bromberg J. S. Jewell C. M. (2018). Designing natural and synthetic immune tissues. *Nat. Mater.* 17 484–498. 10.1038/s41563-018-0077-6 29784994 PMC6283404

[B22] Gosselin E. A. Tostanoski L. H. Jewell C. M. (2017). Controlled release of second generation mTOR inhibitors to restrain inflammation in primary immune cells. *AAPS J.* 19 1175–1185. 10.1208/s12248-017-0089-1 28484962

[B23] Hassett K. J. Cousins M. C. Rabia L. A. Chadwick C. M. O’Hara J. M. Nandi P. (2013). Stabilization of a recombinant ricin toxin A subunit vaccine through lyophilization. *Eur. J. Pharm. Biopharm.* 85 279–286. 10.1016/j.ejpb.2013.03.029 23583494 PMC3797224

[B24] Holzer M. Vogel V. Mäntele W. Schwartz D. Haase W. Langer K. (2009). Physico-chemical characterisation of PLGA nanoparticles after freeze-drying and storage. *Eur. J. Pharm. Biopharm.* 72 428–437. 10.1016/j.ejpb.2009.02.002 19462479

[B25] Izutsu K. Yonemochi E. Yomota C. Goda Y. Okuda H. (2014). Studying the morphology of lyophilized protein solids using X-ray micro-CT: effect of post-freeze annealing and controlled nucleation. *AAPS PharmSciTech* 15 1181–1188. 10.1208/s12249-014-0152-5 24879291 PMC4179649

[B26] Jewell C. M. López S. C. Irvine D. J. (2011). In situ engineering of the lymph node microenvironment via intranodal injection of adjuvant-releasing polymer particles. *Proc. Natl. Acad. Sci. U.S.A.* 108 15745–15750. 10.1073/pnas.1105200108 21896725 PMC3179077

[B27] Keselowsky B. G. Xia C. Q. Clare-Salzler M. (2011). Multifunctional dendritic cell-targeting polymeric microparticles Engineering new vaccines for type 1 diabetes. *Hum. Vaccines* 7 37–44. 10.4161/hv.7.1.12916 21157186 PMC3679212

[B28] Kheddo P. Golovanov A. P. Mellody K. T. Uddin S. van der Walle C. F. Dearman R. J. (2016). The effects of arginine glutamate, a promising excipient for protein formulation, on cell viability: comparisons with NaCl. *Toxicol. In Vitro* 33 88–98. 10.1016/j.tiv.2016.02.002 26873863 PMC4837223

[B29] Lee M. K. Kim M. Y. Kim S. Lee J. (2009). Cryoprotectants for freeze drying of drug nano-suspensions: effect of freezing rate. *J. Pharm. Sci.* 98 4808–4817. 10.1002/jps.21786 19475555

[B30] Mohammadi-Samani S. Taghipour B. (2015). PLGA micro and nanoparticles in delivery of peptides and proteins; problems and approaches. *Pharm. Dev. Technol.* 20 385–393. 10.3109/10837450.2014.882940 24483777

[B31] Nair L. S. Laurencin C. T. (2007). Biodegradable polymers as biomaterials. *Prog. Polym. Sci.* 32 762–798. 10.1016/j.progpolymsci.2007.05.017

[B32] Niu L. Panyam J. (2017). Freeze concentration-induced PLGA and polystyrene nanoparticle aggregation: imaging and rational design of lyoprotection. *J. Control. Release* 248 125–132. 10.1016/j.jconrel.2017.01.019 28093299

[B33] Northrup L. Christopher M. A. Sullivan B. P. Berldand C. (2016). Combining antigen and immunomodulators: emerging trends in antigen-specific immunotherapy for autoimmunity. *Adv. Drug Deliv. Rev.* 98 86–98. 10.1016/j.addr.2015.10.020 26546466

[B34] Park J. Babensee J. E. (2012). Differential functional effects of biomaterials on dendritic cell maturation. *Acta Biomaterialia* 8 3606–3617. 10.1016/j.actbio.2012.06.006 22705044 PMC3970713

[B35] Park J. Gerber M. H. Babensee J. E. (2015). Phenotype and polarization of autologous T cells by biomaterial-treated dendritic cells. *J. Biomed. Mater. Res. Part A* 103 170–184. 10.1002/jbm.a.35150 24616366 PMC4160432

[B36] Pearson R. M. Casey L. M. Hughes K. R. Miller S. D. Shea L. D. (2017). In vivo reprogramming of immune cells: technologies for induction of antigen-specific tolerance. *Adv. Drug Deliv. Rev.* 114 240–255. 10.1016/j.addr.2017.04.005 28414079 PMC5582017

[B37] Shaikh M. V. Kala M. Nivsarkar M. (2017). Formulation and optimization of doxorubicin loaded polymeric nanoparticles using Box-Behnken design: ex-vivo stability and in-vitro activity. *Eur. J. Pharm. Sci.* 100 262–272. 10.1016/j.ejps.2017.01.026 28126560

[B38] Sharp F. A. Ruane D. Claass B. Creagh E. Harris J. Malyala P. (2009). Uptake of particulate vaccine adjuvants by dendritic cells activates the NALP3 inflammasome. *Proc. Natl. Acad. Sci. U.S.A.* 106 870–875. 10.1073/pnas.0804897106 19139407 PMC2630092

[B39] Singh M. ChakRapani A. O’Hagon D. (2007). Nanoparticles and microparticles as vaccine-delivery systems. *Expert Rev. Vaccines* 6 797–808. 10.1586/14760584.6.5.797 17931159

[B40] Sospedra M. Martin R. (2005). Immunology of multiple sclerosis. *Annu. Rev. Immunol.* 23 683–747. 10.1146/annurev.immunol.23.021704.115707 15771584

[B41] Tostanoski L. H. Chiu Y. C. Gammon J. M. Simon T. Andorko J. I. Bromberg J. S. (2016a). Reprogramming the local lymph node microenvironment promotes tolerance that is systemic and antigen specific. *Cell Rep.* 16 2940–2952. 10.1016/j.celrep.2016.08.033 27626664 PMC5024722

[B42] Tostanoski L. H. Gosselin E. A. Jewell C. M. (2016b). Engineering tolerance using biomaterials to target and control antigen presenting cells. *Discov. Med.* 21 403–410. 27355336

[B43] Ulery B. D. Nair L. S. Laurencin C. T. (2011). Biomedical applications of biodegradable polymers. *J. Polym. Sci. B Polym. Phys.* 49 832–864. 10.1002/polb.22259 21769165 PMC3136871

[B44] Vlugt-Wensink K. D. Meijer Y. J. van Steenbergen M. J. Verrijk R. Jiskoot W. Crommelin D. J. (2007). Effect of excipients on the encapsulation efficiency and release of human growth hormone from dextran microspheres. *Eur. J. Pharm. Biopharm.* 67 589–596. 10.1016/j.ejpb.2007.04.011 17540550

