# Supplementary material for: Impact of Excipients on Stability of Polymer Microparticles for Autoimmune Therapy
Source: Front Bioeng Biotechnol. 2021 Feb 11;8:609577. doi: 10.3389/fbioe.2020.609577 (PMC7906284; doi:10.3389/fbioe.2020.609577)
Supplement: Supplementary file 1 [file Data_Sheet_1.docx]

Impact of Excipients on Stability of Polymer Microparticles for Autoimmune Therapy

Supplementary Material

^#^Emily A. Gosselin^1^, ^#^Maeesha Noshin ^1^, Sheneil K. Black^1^, Christopher M. Jewell^1-5*^

^1^Fischell Department of Bioengineering, University of Maryland, College Park, MD, United States

^2^Robert E Fischell Institute of Biomedical Devices, University of Maryland, College Park, MD, United States

^3^United States Department of Veterans Affairs, Baltimore, MD, United States

^4^Department of Microbiology and Immunology, University of Maryland Medical School, Baltimore, MD, United States

^5^Marlene and Stewart Greenebaum Cancer Center, Baltimore, MD, United States

^#^ These authors contributed equally.

*** Correspondence:**Christopher M. Jewell
cmjewell@umd.edu

**Table S1. MPs Reconstitution Assay**

| **MPs Formulation** | **% Resuspended After 10s vortex** |
| --- | --- |
| **MR 0%** | 41.3 |
| **MR Man Enc 1** | 60.6 |
| **MR Man Enc 5** | 67.4 |
| **MR Man Enc 10** | 65.5 |
| **MR Man Ext 1** | 37.2 |
| **MR Man Ext 5** | 66.7 |
| **MR Man Ext 10** | 80.6 |
| **MR Suc Enc 1** | 79.4 |
| **MR Suc Enc 5** | 78.6 |
| **MR Suc Enc 10** | 76.3 |
| **MR Suc Ext 1** | 68.1 |
| **MR Suc Ext 5** | 61.4 |
| **MR Suc Ext 10** | 77.2 |
| **MR Tre Enc 1** | 88.3 |
| **MR Tre Enc 5** | 84.0 |
| **MR Tre Enc 10** | 76.4 |
| **MR Tre Ext 1** | 81.8 |
| **MR Tre Ext 5** | 79.3 |
| **MR Tre Ext 10** | 80.9 |

**Table S2. MPs Rapa and MOG_35-55_ Loading**

| **MPs Formulation** | **Rapa Loading**  **(µg Rapa/ mg formulation)** | **Rapa Loading**  **Efficiency (%)** | **MOG_35-55_ Loading**  **(µg MOG_35-55_/ mg formulation)** | **MOG_35-55_ Loading Efficiency (%)** |
| --- | --- | --- | --- | --- |
| **MR 0** | 14.5 | 51.0 | 6.41 | 43.5 |
| **MR Man Enc 1** | 12.3 | 50.6 | 6.16 | 50.6 |
| **MR Man Enc 5** | 11.5 | 50.0 | 3.75 | 32.5 |
| **MR Man Enc 10** | 11.4 | 49.9 | 2.15 | 18.8 |
| **MR Man Ext 1** | 11.7 | 53.5 | 3.10 | 40.2 |
| **MR Man Ext 5** | 11.9 | 52.6 | 2.06 | 28.9 |
| **MR Man Ext 10** | 9.6 | 52.7 | 1.62 | 22.8 |
| **MR Suc Enc 1** | 11.7 | 46.0 | 3.91 | 40.2 |
| **MR Suc Enc 5** | 11.9 | 50.0 | 2.14 | 28.9 |
| **MR Suc Enc 10** | 9.6 | 39.7 | 1.87 | 22.8 |
| **MR Suc Ext 1** | 10.8 | 43.9 | 4.70 | 38.2 |
| **MR Suc Ext 5** | 6.4 | 46.7 | 2.88 | 41.8 |
| **MR Suc Ext 10** | 3.9 | 43.6 | 1.78 | 39.9 |
| **MR Tre Enc 1** | 17.9 | 59.9 | 3.83 | 25.7 |
| **MR Tre Enc 5** | 16.2 | 54.9 | 2.96 | 20.2 |
| **MR Tre Enc 10** | 15.0 | 51.5 | 2.61 | 17.9 |
| **MR Tre Ext 1** | 11.0 | 41.1 | 4.72 | 35.3 |
| **MR Tre Ext 5** | 6.1 | 50.1 | 2.47 | 40.4 |
| **MR Tre Ext 10** | 3.9 | 42.7 | 1.13 | 24.8 |

**Table S3. MPs Sizing and Polydispersity Index Pre-Lyo, Post-Lyo, and 5 months Post-Lyo**

| **MPs Formulation** | **Pre-Lyo** | | **Post-Lyo** | | **5 mo. Post-Lyo** | |
| --- | --- | --- | --- | --- | --- | --- |
|  | **Diameter (µm)** | **PDI** | **Diameter (µm)** | **PDI** | **Diameter (µm)** | **PDI** |
| **MR 0%** | 3.122 | 0.131 | 3.358 | 0.130 | 4.199 | 0.131 |
| **MR Man Enc 1** | 4.771 | 0.104 | 5.020 | 0.113 | 5.221 | 0.102 |
| **MR Man Enc 5** | 4.930 | 0.130 | 5.156 | 0.130 | 5.774 | 0.107 |
| **MR Man Enc 10** | 4.166 | 0.151 | 3.627 | 0.186 | 4.312 | 0.142 |
| **MR Man Ext 1** | 3.085 | 0.142 | 3.828 | 0.120 | 4.262 | 0.103 |
| **MR Man Ext 5** | 3.074 | 0.132 | 3.235 | 0.129 | 3.905 | 0.099 |
| **MR Man Ext 10** | 3.050 | 0.129 | 3.183 | 0.130 | 3.554 | 0.110 |
| **MR Suc Enc 1** | 6.119 | 0.077 | 5.079 | 0.099 | 5.195 | 0.095 |
| **MR Suc Enc 5** | 4.526 | 0.120 | 4.243 | 0.123 | 4.657 | 0.109 |
| **MR Suc Enc 10** | 4.533 | 0.106 | 4.152 | 0.110 | 4.585 | 0.103 |
| **MR Suc Ext 1** | 3.191 | 0.152 | 2.889 | 0.143 | 3.679 | 0.128 |
| **MR Suc Ext 5** | 3.070 | 0.142 | 2.697 | 0.126 | 3.788 | 0.118 |
| **MR Suc Ext 10** | 3.101 | 0.141 | 2.764 | 0.144 | 3.817 | 0.160 |
| **MR Tre Enc 1** | 5.617 | 0.076 | 5.384 | 0.089 | 7.209 | 0.107 |
| **MR Tre Enc 5** | 4.329 | 0.093 | 4.759 | 0.102 | 5.911 | 0.096 |
| **MR Tre Enc 10** | 4.024 | 0.114 | 4.415 | 0.111 | 4.734 | 0.103 |
| **MR Tre Ext 1** | 2.934 | 0.142 | 2.889 | 0.143 | 3.695 | 0.134 |
| **MR Tre Ext 5** | 2.907 | 0.142 | 2.697 | 0.134 | 3.171 | 0.126 |
| **MR Tre Ext 10** | 2.900 | 0.138 | 2.764 | 0.104 | 4.643 | 0.122 |

**
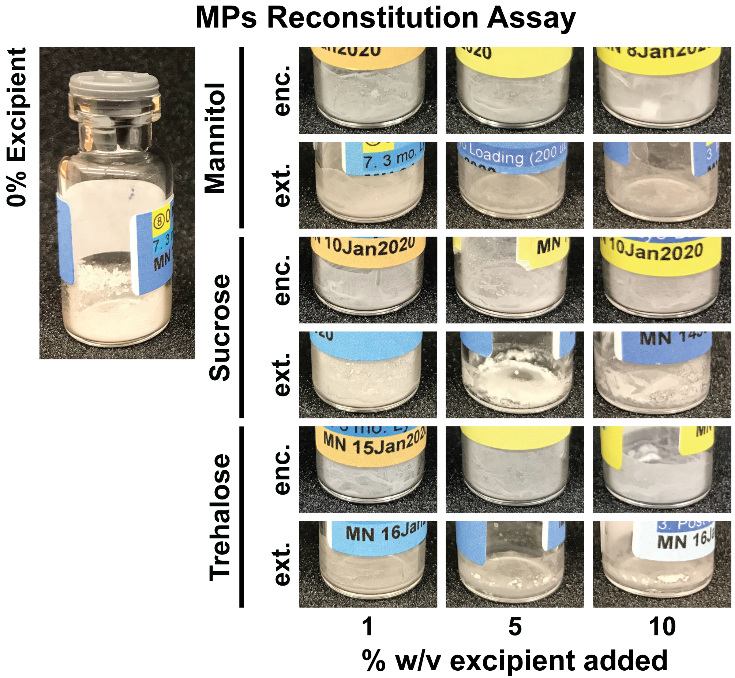
**

**Supplementary Figure 1. MPs lyophilized with excipients are more easily reconstituted.** Images of the remaining MP mass in the original vials after the reconstitution assay and removal of the reconstitution solution.


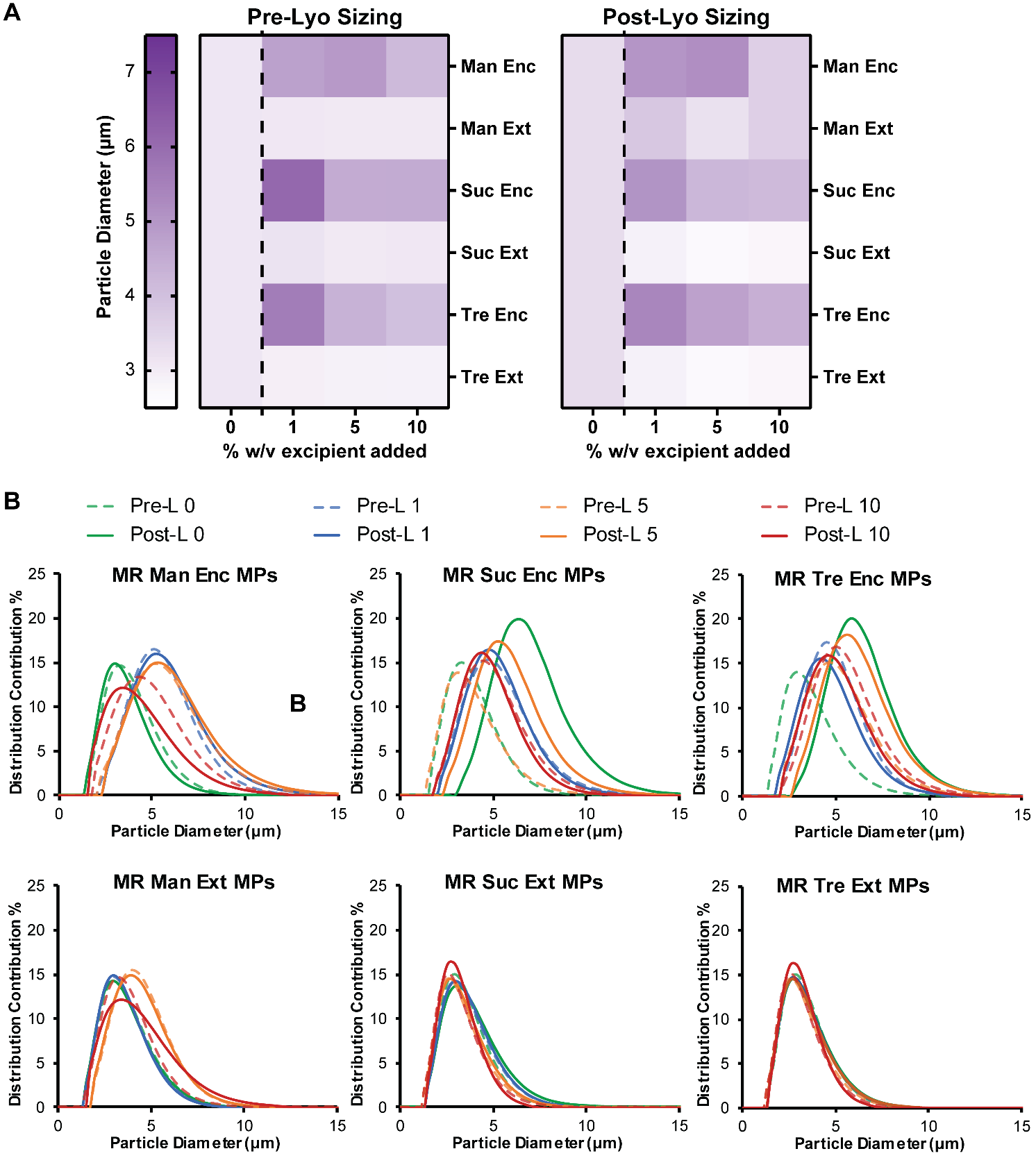


**Supplementary Figure 2. Effect of excipients on MP size before and after lyophilization. (A)** MPs were sized prior to lyophilization and immediately following lyophilization. **(B)** Individual size curves for MOG/Rapa MPs prepared using different excipient conditions.


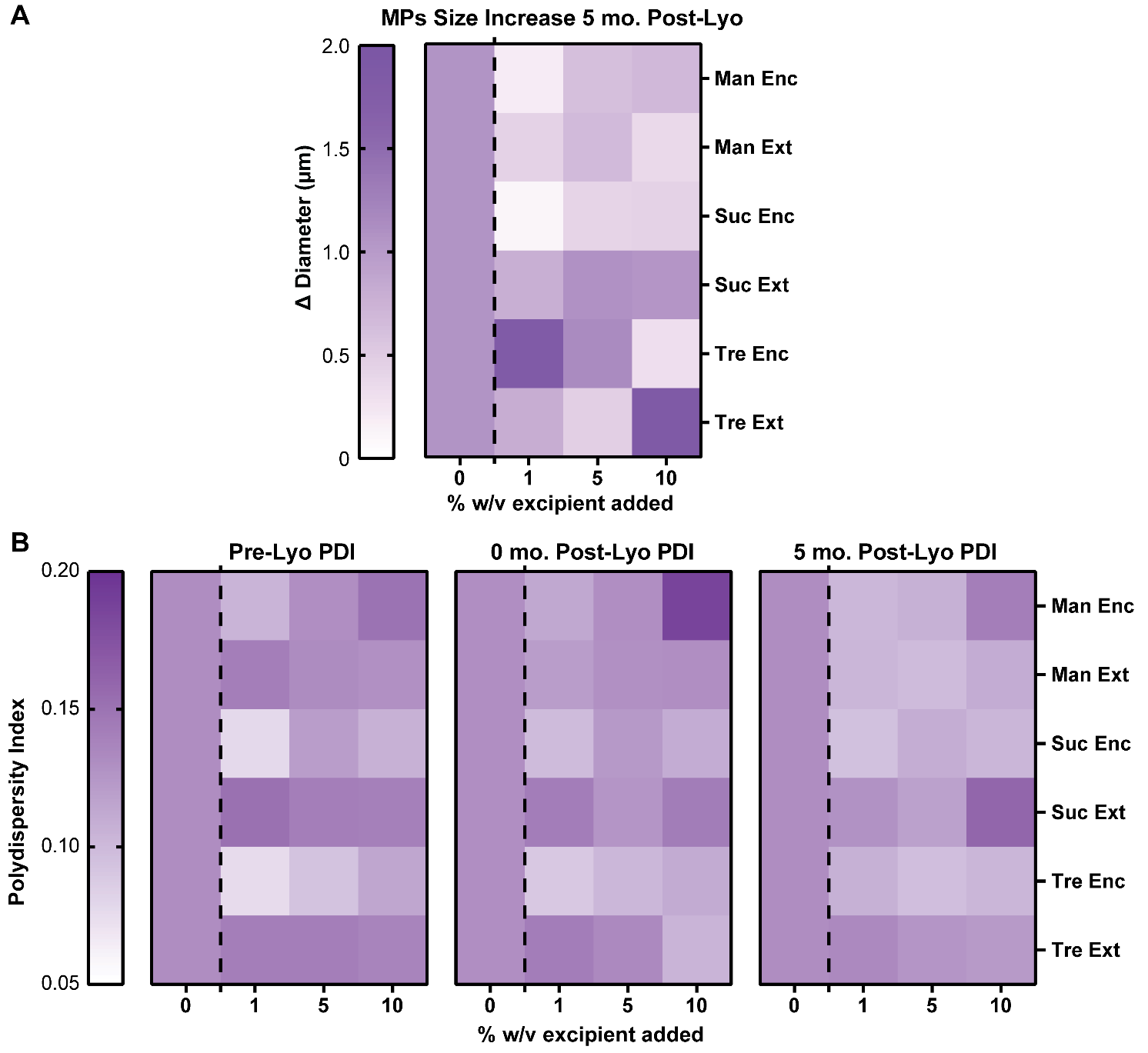


**Supplementary Figure 3. Effect of excipients on MP size and stability during long-term storage.** MPs were sized **(A)** immediately after lyophilization and **(B)** after storage at room temperature for 5 months.

**
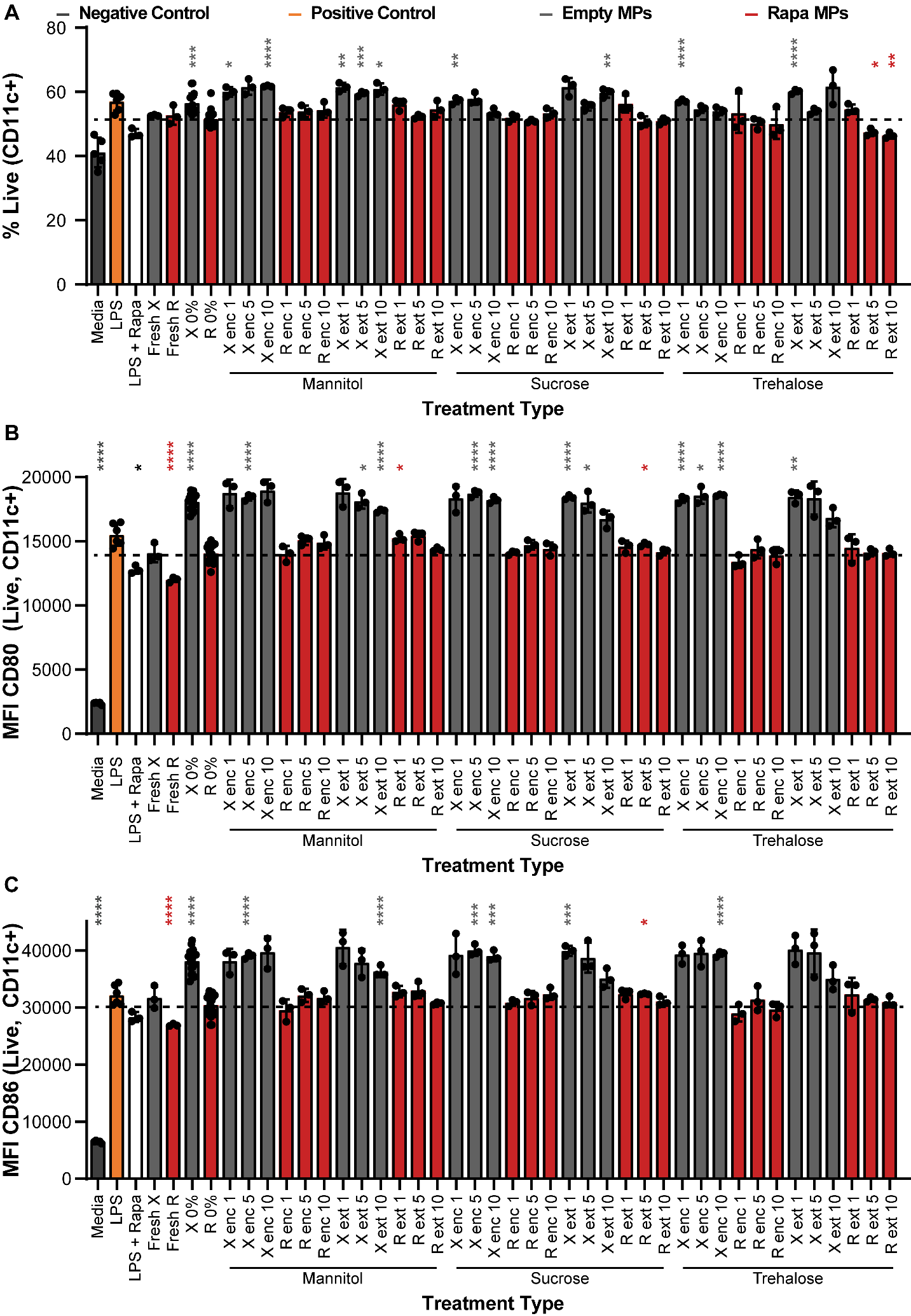
 Supplementary Figure 4. Effect of excipient formulation on DC viability and activation after stimulation.** Primary DCs were collected and cultured for 18h with LPS and Empty or Rapa-loaded MPs. The data with MP treatments is shown in the main text; there are additional controls included here for assay validation. **(A)** Viability of DCs. Activation during treatment, as measured by CD80 **(B)** and CD86 **(C)**. Gray and red asterisks, respectively, indicate a specific Empty MP (gray) or Rapa MP (red) formulation is significant against Rapa MPs without excipient (“R 0%”). For reference, the comparison values are indicated using dashed lines in each panel. For all panels, statistical comparisons were performed using Welch’s ANOVA with a Dunnett’s test for multiple comparisons. (* indicates p ≤ 0.05, ** p ≤ 0.01, *** p ≤ 0.001, **** p ≤ 0.0001).


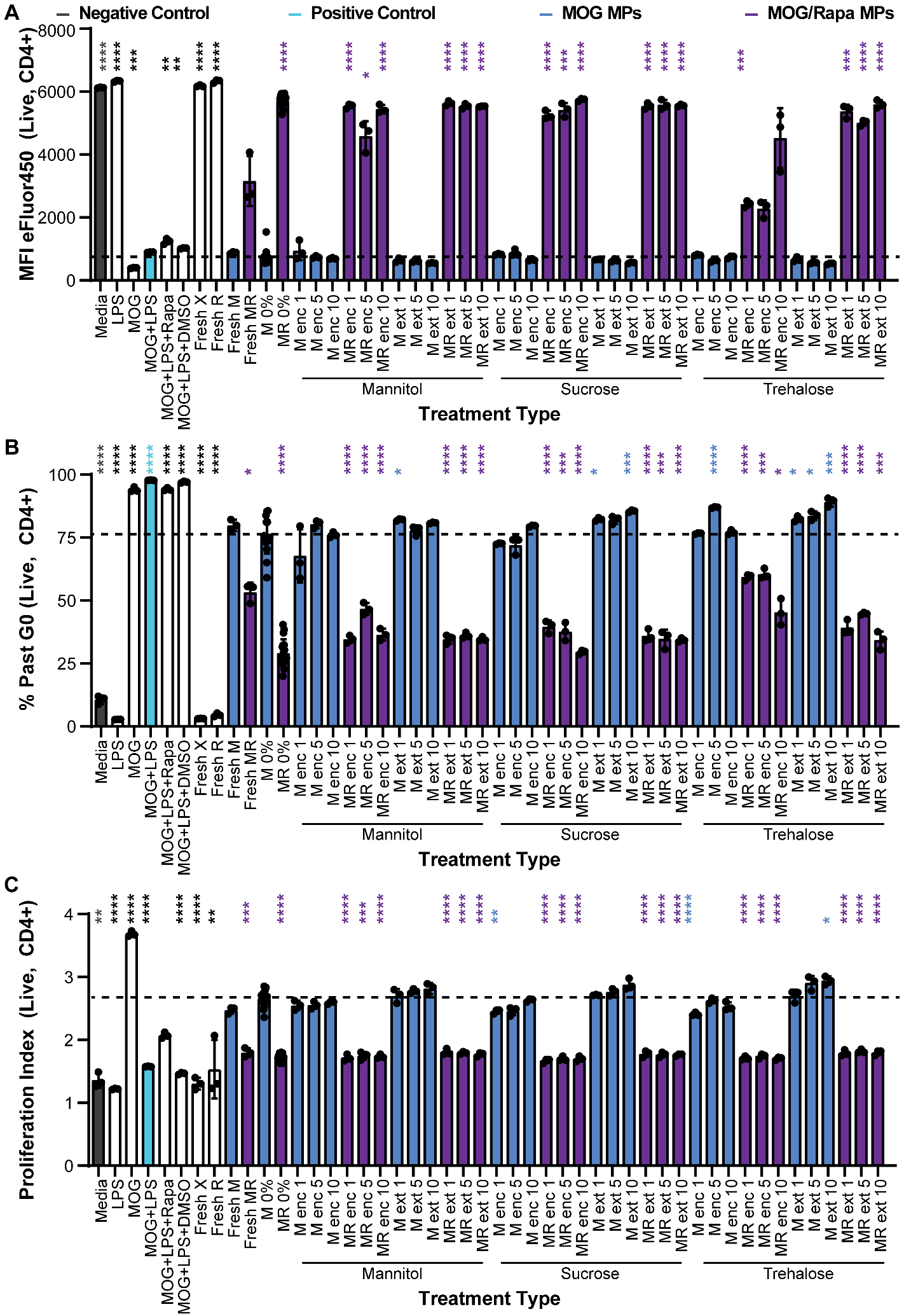


**Supplementary Figure 5. Effect of excipient formulation on MOG-specific T cell expansion.** Primary DCs were collected and cultured for 24h with LPS and either MOG or MOG/Rapa MPs. After 24h, fluorescently-labeled MOG-specific transgenic T cells were added to wells and cultured for 72h to observe differences in proliferation of MOG-specific T cells. The data with MP treatments is shown in the main text; there are additional controls included here for assay validation. **(A)** MFI corresponding to a fluorescent T cell proliferation dye. **(B)** Frequency (%) of proliferation among MOG-specific T cells. **(C)** Proliferation index, reflecting the average number of divisions undergone by proliferating T cells. The color of an asterisk indicates a specific formulation is significant against MOG MPs lyophilized with no excipients (“M 0%”). For reference, the comparison values are indicated using dashed lines in each panel. For all panels, statistical comparisons were performed using Welch’s ANOVA with a Dunnett’s test for multiple comparisons. (* indicates p ≤ 0.05, ** p ≤ 0.01, *** p ≤ 0.001, **** p ≤ 0.0001). The legend in panel A applies to all panels.


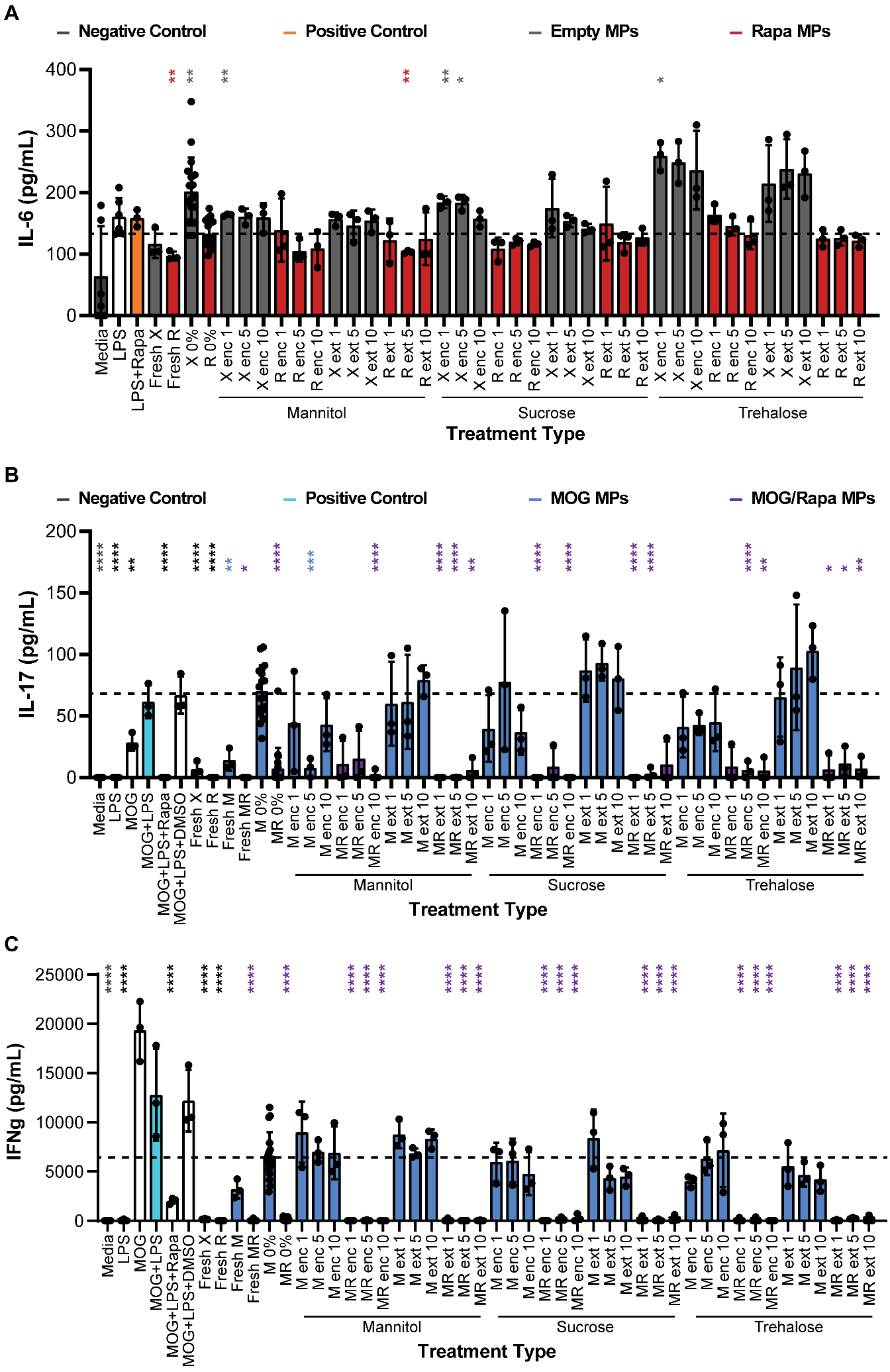


**Supplementary Figure 6. Effect of excipient formulation on inflammatory activity of MOG-specific T cells.** Functional analysis of T cells in DC/MOG-specific T cell co-cultures assessed by ELISA for common inflammatory cytokines, **(A)** IL-6, **(B)** IL-17, and **(C)** IFN- γ. The data with MP treatments is shown in the main text; there are additional controls included here for assay validation. Samples in panel A were compared to Rapa MPs lyophilized with no excipient using Welch’s ANOVA with a Dunnett’s test for multiple comparisons. Samples in panels B and C were compared to MOG MPs lyophilized with no excipient using Welch’s ANOVA with a Dunnett’s test for multiple comparisons. (* indicates p ≤ 0.05, ** p ≤ 0.01, *** p ≤ 0.001, **** p ≤ 0.0001). The color of an asterisk indicates a specific formulation is significant against Rapa MPs without excipient (“R 0%”) in panel A, or MOG MPs without excipient (“M 0%”) in panels B and C. For reference, the comparison values are indicated using dashed lines in each panel. The legend above panel B applies to panels B and C.
